# Key Structural Features of Microvascular Networks Leading to the Formation of Multiple Equilibria

**DOI:** 10.1007/s11538-024-01404-y

**Published:** 2025-01-23

**Authors:** George Atkinson, Yaron Ben-Ami, Philip Maini, Joe Pitt-Francis, Helen Byrne

**Affiliations:** 1 Wolfson Centre for Mathematical Biology, Mathematical Institute, University of Oxford, Woodstock Rd, Oxford, Oxfordshire, OX2 6GG UK; 2 Department of Computer Science, University of Oxford, Parks Rd, Oxford, Oxfordshire, OX1 3QG UK

**Keywords:** Microvascular blood flow, Multiple equilibria, Network redundancy, 74A05, 74A60, 74B20, 74G65, 74K20, 74K25, 74Q05

## Abstract

We analyse mathematical models of blood flow in two simple vascular networks in order to identify structural features that lead to the formation of multiple equilibria. Our models are based on existing rules for blood rheology and haematocrit splitting. By performing bifurcation analysis on these simple network flow models, we identify a link between the changing flow direction in key vessels and the existence of multiple equilibria. We refer to these key vessels as *redundant* vessels, and relate the maximum number of equilibria with the number of redundant vessels. We vary geometric parameters of the two networks, such as vessel length ratios and vessel diameters, to demonstrate that equilibria are uniquely defined by the flow in the redundant vessels. Equilibria typically emerge in sets of three, each having a different flow characteristic in one of the network’s redundant vessels. For one of the three equilibria, the flow within the relevant redundant vessel will be smaller in magnitude than the other two and the redundant vessel will contain few Red Blood Cells (RBCs), if any. For the other two equilibria, the redundant vessel contains RBCs and significant flow in the two available directions. These structural features of networks provide a useful geometric property when studying the equilibria of blood flow in microvascular networks.

**Supplementary Information:**

The online version contains supplementary material available at 10.1007/s11538-024-01404-y.

## Introduction

It is well established that tumour vasculature contains numerous structural abnormalities not found in healthy vasculature, such as: regions of densely packed small vessels and avascular regions (Deane and Lantos 1981), increased microvascular density (Sharma et al 2010; Shieh et al 2009), and the presences of more looped structures (Less et al 1991; Stolz et al 2022). Tumour vasculature has also been described as being structurally and functionally abnormal (Jain 1994). Irregularity of the vasculature arises from increased angiogenesis (formation of new blood vessels) (Forsythe et al 1996; Muz et al 2015; Nagy et al 2009). Geometric irregularity affects the vasculature’s ability to deliver oxygen to the surrounding tumour-microenvironment. It is a potential mechanism driving the formation of hypoxic tumour regions (i.e., low oxygen levels) and, hence, reduced sensitivity to treatments such as radiotherapy Jain (2005). Anti-angiogenic treatments transiently normalise tumour blood flow by pruning capillaries and reducing vessel tortuosity. The associated increases in tissue oxygen levels, have been shown to improve patient responses to treatment (Browder et al 2000; Carmeliet and Jain 2000; Ferrara et al 2004; Jain 2001), and demonstrate the importance of understanding how vascular geometry, blood flow, and hypoxia are related.

It has long been understood that hypoxia within tumours can make cancers more aggressive and harder to treat. Hypoxic tumour regions are associated with increasing resistance to chemo- and radiotherapy (Menegakis et al 2021; Shannon et al 2003; Tan et al 2009), increasing immunosupression and inhibition (Conforti et al 2003; Sitkovsky 2009), and the emergence of highly invasive, metastatic cell phenotypes (Chen et al 2018; Sullivan and Graham 2007). Counter-intuitively, fluctuating oxygen levels in the tumour microenvironment can exacerbate the negative effects of hypoxia. For example, in Chou et al (2012), tumour cells in glioblastomas exposed to cycling hypoxia (short term fluctuations between a normoxic environment and a hypoxic environment) became more resistant to chemotherapy than those exposed to either chronic hypoxia (permanently hypoxic environment) or normoxia. If the mechanisms by which cycling hypoxia emerge can be understood, new treatments that mitigate its adverse effects could be developed.

Theoretical studies of steady blood flow in vascular networks have been used to explore the relationship between irregular vasculature and hypoxia within tumours (Bernabeu et al 2020; Sweeney et al 2018). These studies typically assume that a single stable flow equilibrium exists. However, as demonstrated by Gardner et al (2010) and Karst et al (2017), even simple networks can admit multiple equilibria and periodic solutions (Ben-Ami et al 2022; Karst et al 2015). In particular, in a recent study of blood flow through a simple, triangular network, a link was established between its structural features and the existence of oscillatory solutions and multiple equilibria Ben-Ami et al (2022). The authors hypothesise that similar oscillations may cause cycling hypoxia in tumours.

In this paper, we extend the work carried out by Gardner et al (2010) by studying the key structural components of the triangle network and a network created by the addition of a single vessel to the triangle network, which we refer to as the extended-triangle network. By studying two simple networks which share structural properties, we identify the presence of vessels known as *redundant* vessels as the key geometric property of vascular networks leading to the emergence of multiple equilibria. We perform a systematic bifurcation analysis in which we vary common vessel length ratios and diameters to show that changing flow direction in the redundant vessels is consistently associated with multiple equilibria, regardless of the length ratios or vessel diameters. This observation explains how multiple equilibria may form and can also be used to identify vascular networks which may admit multiple equilibria. Such understanding is important to the study of the formation of hypoxia within tumours. In particular, a better understanding of the formation of multiple equilibria may also enable us to uncover the mechanisms by which cycling hypoxia emerges.

In Sect. 2, we describe the principles of blood flow in the microvasculature and introduce the notation we use to represent vascular networks. In Sect. 3 we explain how the network equations associated with a network are constructed, introduce the networks studied in this paper, and the methods we use to solve the network equations. In Sect. 4 we present our results and demonstrate how network redundancy leads to the existence of multiple equilibria. We summarise our conclusions in Sect. 5 where we also discuss the implications of our work.

## Blood Flow in the Microcirculation

In this section, we introduce the physical principles that we use to model blood flow through a network of blood vessels. We start in Sect. 2.1 by explaining how we model blood flow in a single vessel. Then, in Sect. 2.2, we introduce the dependent variables, parameters and associated notation that we use when simulating blood flow through vascular networks. Finally, in Sects. 2.3 and 2.4, we describe the conservation laws and splitting rules that are used to construct the governing equations.

### Steady Flow in a Single Vessel

Blood transport in a microvessel is often viewed as a Newtonian fluid, with a constant viscosity, $\mu$, flowing according to Poiseuille’s law, within a rigid cylinder, with no-slip conditions imposed on the walls of the cylinder (Hagenbach 1860; Poiseuille 1846). Under these assumptions, we have that:

1 $$\begin{aligned} Q = \frac{\Delta P}{R}, \end{aligned}$$

where *Q* is the volumetric flow rate through the vessel, $\Delta P$ is the pressure drop across the length of the vessel, and *R* is its hydraulic resistance. For a rigid pipe, the hydraulic resistance is defined by the following expression:

2 $$\begin{aligned} R = \frac{128 L\mu }{\pi D^4}, \end{aligned}$$

where $L$ and $D$ denote the vessel’s length and diameter, respectively.

In practice, blood contains cells and proteins suspended in plasma. The effect of these suspended particles on the rheology can typically be ignored, with the exception of Red Blood Cells (RBCs). Pries et al (1992) performed multiple experiments on RBC suspensions in glass tubes to establish an empirical relationship between the density of RBCs and the resistance of the blood. By measuring the flow in a glass tube for a controlled pressure drop, and counting the number of RBCs travelling through the tube, they determined the resistance to flow as a function of the haematocrit (the ratio of the volumetric flow of the RBCs to the volumetric flow of the blood). Then by assuming Poiseuille’s law, they derived a function for the blood’s effective viscosity. Pries et al (1994) later used *in vivo* experiments to obtain a more accurate expression for the viscosity:

3 $$\begin{aligned} \mu (H,D) = \mu _p \Bigg [1 + (\mu _{45} - 1)\frac{(1 - H)^C - 1}{0.55^C - 1}\bigg (\frac{D}{D- 1.1}\bigg )^2\Bigg ]\bigg (\frac{D}{D- 1.1}\bigg )^2, \end{aligned}$$

where $D$ is the vessel diameter in $\mu \text {m}$, *H* is the haematocrit in the vessel, $\mu _p$ is the viscosity of the plasma, and $\mu _{45}$, the relative apparent blood viscosity when $H = 0.45$, is defined as follows:

4 $$\begin{aligned} \mu _{45} = 6\exp (-0.085 D) + 3.2 -2.44\exp (-0.06 D^{0.645}), \end{aligned}$$

In equation (3), the coefficient *C* is defined as follows:

5 $$\begin{aligned} C = (0.8 + \exp (-0.075 D)) \Bigg (-1 + \frac{1}{1 + 10^{-11}D^{12}}\Bigg ) + \frac{1}{1 + 10^{-11}D^{12}}. \end{aligned}$$

Then, the resistance in the vessel is given by:

6 $$\begin{aligned} R(H,D,L) = \frac{128 L\mu (H,D)}{\pi D^4}. \end{aligned}$$

The pressure difference across a pipe is the difference between the inlet and outlet pressure. Therefore, steady flow in a pipe is constant flow such that the volumetric flow is described with the following equation:

7 $$\begin{aligned} Q = \frac{(P_{in}-P_{out})}{R(H,D,L)}, \end{aligned}$$

where $P_{in}$ and $P_{out}$ are the pressures at the two ends of the vessel.

### Vascular Networks

In this section, we define the notation we use to represent vascular networks and introduce the concept of a redundant vessel by exploiting properties of directed networks. We denote by $\mathcal {N}= \{N,E\}$ a vascular network where edges *E* represent blood vessels and nodes *N* represent vessel junctions, inlets or outlets. By convention, inlet and outlet nodes have degree 1. All other internal nodes represent vessel junctions and must have degree 3 or more (two vessels with a shared node of degree 2 are considered equivalent to a single vessel). It will sometimes be convenient to distinguish between in and out degree of a node to specify the blood flow direction. For example, a node with and in degree of 1 and an out degree of 2, has one vessel flowing into the node, and two vessels flowing out of the node.

Dependent variables and model parameters are indexed by the relevant nodes or edges. For example, $P_x$ and $P_y$ denote the pressures at nodes *x* and *y*, and $Q_{(x,y)}, H_{(x,y)},L_{(x,y)}$ and $D_{(x,y)}$ denote the volumetric flow, haematocrit, length and diameter in vessel (*x*, *y*) respectively. If either node *x* or *y* is a boundary node then the associated pressure is a parameter, and denoted with a bar for clarity. For example, if node *x* is a boundary node, then $\overline{P}_x$ is the prescribed pressure there. If *x* is an inlet node, and (*x*, *y*) an inlet vessel, then the parameter $\overline{H}_{(x,y)}$ represents the associated inlet haematocrit.

Rewriting the hydraulic resistance Eq. (6) in terms of the network parameters and variables yields the following equation:

8 $$\begin{aligned} R_{(x,y)} = \frac{128 L_{(x,y)} \mu (H_{(x,y)},D_{(x,y)})}{\pi D_{(x,y)}^4}. \end{aligned}$$

Further, using the network variables, Poiseuille’s law can be written as follows:

9 $$\begin{aligned} Q_{(x,y)} = \frac{P_x - P_y}{R_{(x,y)}(H_{(x,y)},D_{(x,y)},L_{(x,y)})}. \end{aligned}$$

For a vessel $(x,y) \in E$, the blood flow direction is dictated by the values of the pressure variables, $P_x$ and $P_y$. However, this does not mean that the blood flow direction in all vessels is restricted to one direction. The blood flow in all inlet nodes must be directed away from the inlet node and the blood flow in all outlet nodes must be directed towards the outlet node. Since blood flows from higher to lower pressures, the values of the inlet pressures are always larger than the values of the outlet pressures. It is technically possible for inlet nodes to switch to outlet nodes (or vice versa) if the boundary pressures change but we do not consider this scenario here.

The theory blood flow direction in non-inlet or outlet vessels may change as the network parameters vary. In practice, however, the flow direction is constrained by the network geometry. Consider, for example, a vascular network $\mathcal {N}= \{N,E\}$ with *m* vessels and *n* nodes. Let $\mathcal {G}_{\mathcal {N}}$ be the set of all directed networks based on network $\mathcal {N}$ for which the direction of flow can be induced by a distribution of nodal pressures. One consequence of these criteria is that all networks $G \in \mathcal {G}_{\mathcal {N}}$ must be acyclic because flow loops are not possible. A loop in a network is any path that starts and ends at the same node. Loops are not possible in vascular networks because a flow loop contradicts the assumption that fluid flows from high to low pressure. Consider a sequence of *k* nodes, $\{u_i\}_{i=1}^k$, such that $u_1 = x, u_k = x,$ and $u_i \ne x$ for $1< i< k$. For this loop to belong to a network $G \in \mathcal {G}_{\mathcal {N}}$, this would imply that:

10 $$\begin{aligned} P_x> P_{u_1}>....> P_{u_{k-1}} > P_x. \end{aligned}$$

This is clearly a contradiction. Therefore, loops of flow cannot exist. An important consequence of this property is that the number of possible flow directions in a network is limited by its geometry.

#### Definition 1

(Redundant vessels) Consider a network $\mathcal {N}= \{N,E\}$. Let $\mathcal {G}_{\mathcal {N}}$ denote the set of all acyclic directed networks of $\mathcal {N}$ such that all inlet nodes have an out degree of 1, all outlet nodes have an in degree of 1, and all interior nodes have an in and out degree of at least 1. If $G_1=\{N,E_1\} \in \mathcal {G}_{\mathcal {N}}$ and $G_2=\{N,E_2\} \in \mathcal {G}_{\mathcal {N}}$ such that a vessel $(x,y) \in E, (x,y) \in E_1$ and $(y,x) \in E_2$, then vessel (*x*, *y*) is a redundant vessel.

The term “redundant vessel" was first used by Ben-Ami et al (2022) to describe a vessel for which a solution existed with zero flow in that specific vessel. However, the key topological property of the network that led to multiple equilibria and instability of the blood flow was the fact that the flow direction was not fixed in this vessel. Therefore, the redundant vessel introduced by Ben-Ami et al (2022) is consistent with Definition 1.

If a vessel is *not* a redundant vessel, then we term it a “fixed vessel”. From now on, we use triangular bracket notation to denote redundant vessels. For example, if there is a redundant vessel connecting nodes *x* and *y*, then we denote it by $\langle x,y \rangle$. Round brackets will be used to denote fixed vessels.

### Conservation Laws

**Fig. 1:**
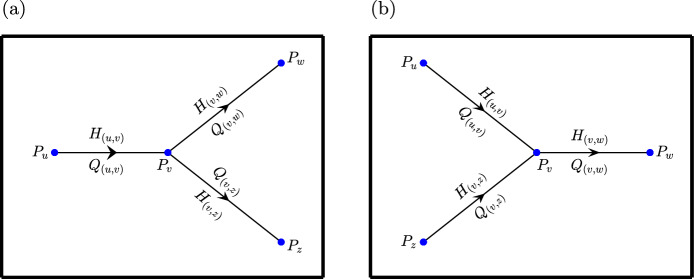
Schematic diagrams illustrating the two basic units which represent only two possible arrangements of flow directions in and out of a vessel junction. **a** Bifurcation unit, with a single input and two outlet vessels; **b** Convergence unit, with two inputs and a single outlet. In each diagram, pressures are defined at the nodes ($P_u,P_v,P_w$ and $P_z$), volumetric flow rates and haematocrits in each vessel are denoted by $Q_{(u,v)}, Q_{(v,w)}$ and $Q_{(v,z)}$, and $H_{(u,v)}, H_{(v,w)}$ and $H_{(v,z)}$ respectively. Arrows indicate flow directions

A fundamental principle underpinning mathematical models of blood flow in the microcirculation is mass conservation at vessel junctions. This principle applies to RBCs and to blood as a whole, here taken to be plasma and RBCs. Conservation of flow can be intuitively described as “flow in equals flow out." For example, for a node with degree 3, only two flow configurations are possible, as shown in Fig. 1. We represent conservation of flow and RBCs for the two flow configurations in Fig. 1 by the following equations:

11 $$\begin{aligned} Q_{(u,v)} + Q_{(w,v)} + Q_{(z,v)}&= 0, \end{aligned}$$

12 $$\begin{aligned} Q_{(u,v)} H_{(u,v)} + Q_{(w,v)} H_{(v,w)} + Q_{(z,v)} H_{(v,z)}&= 0. \end{aligned}$$

### Splitting Rules

At a flow convergence (see Fig. 1b), conservation of RBCs is sufficient to uniquely determine the value of $H_{(v,w)}$ if the inlet flows and haematocrits are known. This also applies to flow convergences of three or more vessels flowing into a single out vessel. At a junctions with two or more outflowing vessels like the flow bifurcation in Fig. 1a, conservation of RBCs is insufficient to determine the haematocrit values in the daughter vessels: splitting rules are introduced to uniquely determine the outlet haematocrits.

Splitting rules are used throughout the literature to define the ratio of the volumetric flow of RBCs in a daughter vessel to that in the parent vessel (Bernabeu et al 2020; Fenton and Zweifach 1981; Klitzman and Johnson 1982; Pries et al 1989, 1990; Pries and Secomb 2005). While there is no consensus on how this ratio should be specified, we use splitting rules that are functions of the flow ratio $r=Q_{(v,w)}/Q_{(u,v)}$, and the parent haematocrit, $H_{(u,v)}$(Bernabeu et al 2020; Klitzman and Johnson 1982; Pries et al 1989, 1990; Pries and Secomb 2005). For example, Pries et al (1989) proposed the following functional form for $\psi _{(v,w)}$ for the (*v*, *w*) vessel of the flow bifurcation in Fig. 1a:

13 $$\begin{aligned} \psi _{{(v,w)}}(r) = {\left\{ \begin{array}{ll} 0 & \text { if } r < X_0 \\ 1 & \text { if } r > 1-X_0 \\ \frac{e^A(r-X_0)^\rho }{e^A(r-X_0)^\rho + (1-r-X_0)^\rho } & \text {if }X_0 \le r \le 1-X_0 \end{array}\right. } \end{aligned}$$

The behaviour of this splitting rule is determined by the $X_0,A,$ and $\rho$ coefficients. One example for these coefficients suggested by Pries et al (1990) is as follows:

14 $$\begin{aligned} A&= -\frac{6.96}{D_{(u,v)}}\log \bigg (\frac{D_{(v,w)}}{D_{(v,z)}}\bigg ), \end{aligned}$$

15 $$\begin{aligned} \rho&= 1 + 6.98\frac{1-H_{(u,v)}}{D_{(u,v)}}, \end{aligned}$$

16 $$\begin{aligned} X_0&=\frac{0.4}{D_{(u,v)}}. \end{aligned}$$

We refer to this splitting rule as the Pries 1990 splitting rule to distinguish it from an alternative rule proposed by the same authors in which different functional forms were used for the coefficients $A,\rho$ and $X_0$ (Pries and Secomb 2005). When the blood flow is at a steady-state, the following equality holds:

17 $$\begin{aligned} \psi _{(v,w)}(r) = \frac{Q_{(v,w)} H_{(v,w)}}{Q_{(u,v)} H_{(u,v)}}. \end{aligned}$$

While, in theory, splitting rules can be defined for junctions involving any number of vessels, they typically focus on junctions involving three vessels. Henceforth we restrict attention to networks for which the maximum node degree is three.

## Network Equations and Methods

Drawing on the principles introduced in Sect. 2, we now introduce the equations governing blood flow through vascular networks and then describe the methods we use to solve the equations. In Sect. 3.1 we introduce the governing equations for a generic network. Then, in Sect. 3.2 we describe the networks we use to study the effect of network redundancy on the number of equilibria. We also non-dimensionalise the network equations, and discuss the parameter values we use vary to determine the relationship between multiple equilibria and the redundant vessels. In Sect. 3.3 we explain how numerical continuation is used to solve the network equations and to identify bifurcation points as key network parameters are varied.

### Network Equations

In this section, we explain how we construct the steady-state equations for the dependent variables associated with blood flow through a vascular network. Pressure is prescribed at network inlets and outlets and haematocrit is prescribed at inlets. Governing equations for the nodal pressures, vessel flows and haematocrit are formulated using the network notation introduced in Sect. 2.2. This description of blood flow in a network is similar to that of electricity in a circuit where volumetric flow Eq. (9), pressure difference, and hydraulic resistance Eq. (6) are analogous to current, voltage, and electrical resistance, respectively (Pries and Secomb 2008). The solution to the steady-state equations must satisfy the conservation laws and splitting rules defined by Eqs. (11-13) at every node. We begin by describing the construction of the network equations for any vascular network, $\mathcal {N}$.

In addition to the network equation obeying the conservation laws, we must also have an equation for every pressure and haematocrit variable of the network. Each flow variable in the network can be replaced with the expression on the right hand side of Eq. (9). Therefore, the flows are replaced by functions of pressures and haematocrits. For both a convergence and bifurcation unit in Fig. 1 the equation associated with $P_v$ is the same as the conservation of flow equation Eq. 11):

18 $$\begin{aligned} Q_{(u,v)}+Q_{(w,v)}+Q_{(z,v)}=0. \end{aligned}$$

The equation associated with $H_{(v,w)}$ can take one of two forms, depending if (*v*, *w*) is a daughter vessel of a bifurcation unit (Fig. 1a) or the outflowing vessel of a convergence unit (Fig. 1b). If (*v*, *w*) is the outflowing vessel for a convergence unit, then the equation associated with $H_{(v,w)}$ is the same as the conservation of RBCs equation Eq. (12):

19 $$\begin{aligned} Q_{(u,v)}H_{(u,v)} + Q_{(z,v)}H_{(z,v)} + Q_{(w,v)}H_{(w,v)}=0. \end{aligned}$$

If (*v*, *w*) is the daughter vessel of a bifurcation unit, then the equation associated with $H_{(v,w)}$ is:

20 $$\begin{aligned} \psi _{(v,w)}(Q_{(v,w)}/Q_{(u,v)}, H_{(u,v)})Q_{(u,v)}H_{(u,v)} - Q_{(v,w)}H_{(v,w)}=0, \end{aligned}$$

where $\psi _{(v,w)}$ is the splitting rule defined in Eq. (13). Unless otherwise specified, $\psi _{(v,w)}$ will refer to the Pries 1990 splitting rule defined using the coefficients in Eq. (14-16).

For a network $\mathcal {N}$, we define a set of network equations by $\textbf{F}(\textbf{H},\textbf{P}) = \textbf{0}$, where $\textbf{P}=[P_{v \in N}]$ is the vector of pressure variables, $\textbf{H}=[H_{(u,v) \in E}]$ is the vector of haematocrit variables, and:

21 $$\begin{aligned} \textbf{F}(\textbf{H},\textbf{P})=\left[ \begin{array}{c} \left[ F_{v \in N}\right] \\ \left[ F_{(v,w) \in E}\right] \end{array}\right] , \end{aligned}$$

such that $F_v$ is the left-hand side of Eq. (18), and $F_{(v,w)}$ is the left-hand side of Eqs. (19) or (20). Therefore, solutions to the following system of equations $\textbf{F}(\textbf{H},\textbf{P}) = \textbf{0}$ satisfy the conservation laws and splitting rules for a network, and every variable has a corresponding equation. The flow direction in certain vessels may change as the network parameters vary, corresponding to a change in the form of $F_{(v,w)}(H,P)$ between Eqs.(19) and (20), but the formulation remains consistent with the conservation laws and splitting rules.

### Network Geometries

**Fig. 2:**
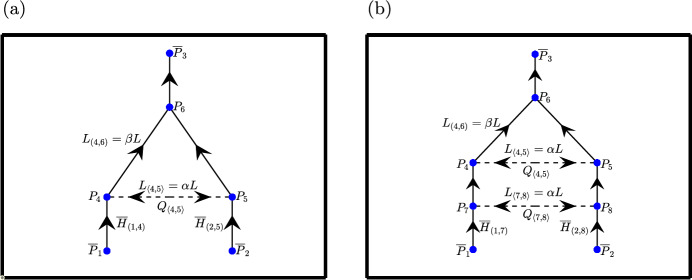
Schematic diagrams of **a** the triangle network and **b** the extended-triangle network. The redundant vessels (Definition 1) are represented by dashed lines. The flow in the redundant vessels, the nodal pressures, inlet haematocrits and relevant vessel lengths are labelled on both diagrams. All other variables and parameters can be inferred from the node numbering. The arrows indicate the possible flow directions in each vessel. All unlabelled vessel lengths have default length of $L$

The aim of this paper is to understand the relationship between the number of steady-state solutions and the number of redundant vessels in a network. As such, it is important to study more than one network. It is, however, difficult to compare networks with different geometries. In order to obtain a meaningful comparison, we study two networks which share common features, but have different numbers of redundant vessels: the triangle network, which has one redundant vessel (see Fig. 2a) and the extended-triangle network, which has two redundant vessels (see Fig. 2b). The triangle network was studied by Gardner et al (2010), and is one of the simplest networks that admits multiple equilibria. The extended-triangle network is the simplest addition to the triangle network which adds an additional redundant vessel. Therefore, comparing the number of equilibria of the triangle network with the extended-triangle network will clearly demonstrate the impact of the level of network redundancy on the multiplicity of solutions.

Direct comparison of the triangle and extended-triangle networks is not possible because they have different parameters and variables. Therefore, in what follows, we will compare variables which provide similar information about the equilibria and vary parameters which have a similar effect on the network equilibria. Given our focus on the impact of vessel redundancy on network equilibria, we compare the equilibria of the two networks by comparing the flow variables in the redundant vessels ($Q_{\langle 4,5 \rangle }$ for the triangle network, and $Q_{\langle 4,5 \rangle }$ and $Q_{\langle 7,8 \rangle }$ for the extended-triangle network).

Many network parameters could be used to demonstrate differences between the two networks. We focus on two vessel length ratios, $\alpha$ and $\beta$, because they have a similar effect on the flow in both networks. $L$ denotes the reference length for both networks, which is the default length for most vessels and we define the length ratios $\alpha$ and $\beta$ with respect to this reference length in the following way:

22 $$\begin{aligned} \alpha = \frac{L_{\langle 4,5 \rangle }}{L} \end{aligned}$$

in the triangle network and

23 $$\begin{aligned} \alpha = \frac{L_{\langle 4,5 \rangle }}{L} = \frac{L_{\langle 7,8 \rangle }}{L} \end{aligned}$$

in the extended-triangle network. Additionally,

24 $$\begin{aligned} \beta = L_{(4,6)}/L, \end{aligned}$$

for both networks. The above definitions of $\alpha$ and $\beta$ for the triangle network are identical to those used by Gardner et al (2010). As the resistance to flow Eq. (6) is proportional to $L$, the ratio $\alpha$ affects the hydraulic resistance in all redundant vessels.

The ratio $\beta$ sets the length of one of the fixed vessels in the triangle subnetwork common to both networks. This subnetwork comprises nodes 4, 5 and 6, and vessels (4, 6), (5, 6) and $\langle 4,5 \rangle$. We assume equal inlet pressures and haematocrits for both networks (see Fig. 2), and so $\beta$ determines the degree of network asymmetry. All other vessels in both networks are of length $L$.

The governing equations for both networks can be constructed from the schematics in Fig. 2 and by following the steps outlined in Sect. 3.1. Statements of the governing equations for both networks can be found in Supplementary Material S1.

#### Dimensionless Network Equations

To facilitate comparison of the two networks described in Fig. 2, it is important that the network equations and variables are dimensionless. The haematocrit variables are already defined as ratios and so their values are always between 0 and 1, but the volumetric flows depend on network parameters such as boundary pressures. Therefore, we need to first convert the flow variables into dimensionless quantities to compare the equilibria of the two networks. Conservation of flow guarantees that the volumetric flow in any vessel is always less than the total flow into a network. Therefore we nondimensionalise volumetric flow in vessel (*x*, *y*) with respect to the total flow into the network:

25 $$\begin{aligned} \hat{Q}_{(x,y)} = \frac{Q_{(x,y)}}{Q_{in}}. \end{aligned}$$

Here, $\hat{Q}_{(x,y)}$ is the dimensionless flow, and $Q_{in}$ is the total flow into the network. For the triangle network, $Q_{in} = Q_{(1,4)} + Q_{(2,5)}$; for the extended-triangle network, $Q_{in} = Q_{(1,7)} + Q_{(2,8)}$. With $0 \le |\hat{Q}_{(x,y)}| \le 1$ for all vessels, it is easier to make direct comparison of the flows in the two networks.

An additional advantage of using dimensionless flows is that they are independent of the reference vessel length, $L$. As such, we can study the effect of varying the vessel length ratios $\alpha$ and $\beta$ (see Eqs. (22–24)), without reference to $L$.

If the flow variables on the left-hand side of Eqs. (11) and (12) are replaced by dimensionless variables, mass is still conserved at every node:

$$\begin{aligned} (\underbrace{Q_{(u,v)} + Q_{(w,v)} + Q_{(z,v)}}_{=0})/Q_{in}&=0, \\ (\underbrace{Q_{(u,v)}H_{(u,v)} + Q_{(w,v)}H_{(w,v)} + Q_{(z,v)}H_{(v,z)}}_{=0})/Q_{in}&=0. \end{aligned}$$

If we replace the flows in Eq. (20) with the dimensionless flow, the equations dictating RBC splitting at flow bifurcations are unaffected by the change in variables:

26 $$\begin{aligned} \psi _{(v,w)}\bigg (\frac{(Q_{(v,w)}/Q_{in})}{(Q_{(u,v)}/Q_{in})}, H_{(u,v)}\bigg ) \frac{Q_{(u,v)}}{Q_{in}}H_{(u,v)} - \frac{Q_{(v,w)}}{Q_{in}}H_{(v,w)}&=0, \end{aligned}$$

27 $$\begin{aligned} (\psi _{(v,w)}(Q_{(v,w)}/Q_{(u,v)}, H_{(u,v)})Q_{(u,v)}H_{(u,v)} - Q_{(v,w)}H_{(v,w)})/Q_{in}&=0. \end{aligned}$$

As the conservation laws and flow ratios are unaffected by normalisation, we can define the network equations in terms of the flow ratios without changing the solutions to the network equations. Henceforth, we drop the hat notation in Eq. (25) and all volumetric flows are normalised.

In addition to normalising the flows, we will also use dimensionless quantities to describe the pressures and vessel diameters of the network. The pressures are normalised by replacing pressure the variable $P_v$ with $(P_v-\overline{P}_3)/(\overline{P}_1+\overline{P}_2)$, and the boundary pressure parameters $\overline{P}_v$ with $(\overline{P}_v-\overline{P}_3)/(\overline{P}_1 + \overline{P}_2)$. The normalised pressures do not change the equilibria of a network for normalised volumetric flow variables.

Unlike vessel lengths and network pressures, vessel diameters cannot be replaced by diameter ratios. This is because changing the scale of the diameters changes the behaviour of the viscosity equation (see Eq. 3), and the coefficients of the splitting rule Eq. (14–16). Therefore, we normalise the diameter by units of $1 \mu \text {m}$.

#### Network Parameters

When performing bifurcation analysis, unless otherwise stated, we use the following default parameter values for both networks (see Fig. 2):

$$\begin{aligned} \overline{P}_1&= \overline{P}_2 = 0.5, \;\;D= 10 , \;\; \alpha = 0.1, \;\; \beta = 1,\\ \overline{H}_{(1,7)}&= \overline{H}_{(2,8)} = \overline{H}_{(1,4)} = \overline{H}_{(2,5)} = \overline{H}_{in}=0.45, \end{aligned}$$

where $D$ is the diameter of all network vessels, and $\alpha$ and $\beta$ are the vessel length ratios defined in Eqs.(22–24). Thus, we impose symmetric inlet conditions, and fix a common value for all vessel diameters. Later on, by changing the value of $\beta$, we emphasise the effect of network asymmetry (recall that network asymmetry increases as $|\beta - 1|$ increases). We do not choose values for the reference length $L$ because the value does not effect the values of the dimensionless parameters or variables.

### Numerical Continuation

Given the nonlinearity of the network equations, solving the network equations, and finding instances of multiple equilibria is difficult. Therefore, we use numerical continuation to find an initial equilibrium of the network equations, and to generate bifurcation diagrams of the equilibria. To find an equilibrium of the system of network equations, we use a homotopy function:

28 $$\begin{aligned} \textbf{h}(\textbf{H},\textbf{P},\lambda ) = \textbf{F}(\textbf{H},\textbf{P})(1-\lambda ) +\textbf{G}(\textbf{H},\textbf{P})\lambda , \end{aligned}$$

where $\textbf{F}$ is the set of network equations described in Sect. 3.1, $\textbf{G}$ is a starting system of equations of our choosing, and $\lambda$ is a parameter between 0 and 1. If the solution to:

29 $$\begin{aligned} \textbf{h}(\textbf{H},\textbf{P},1) = \textbf{G}(\textbf{H},\textbf{P})={\textbf {0}}, \end{aligned}$$

is known, then a solution to:

30 $$\begin{aligned} \textbf{h}(\textbf{H},\textbf{P},0) = \textbf{F}(\textbf{H},\textbf{P})={\textbf {0}}, \end{aligned}$$

can be found by tracking the solution from $\lambda =1$ to $\lambda =0$. $\lambda$ is tracked by generating a Davidenko differential equation for $\textbf{h}$, for which $\lambda$ is the independent variable and $(\textbf{H},\textbf{P})$ are the dependent variables. This equation is derived by taking the first derivative of $\textbf{h}=\textbf{0}$ with respect to $\lambda$ (Allgower and Georg 2003). Then tracking the solution as $\lambda$ is varied is equivalent to numerically solving the Davidenko differential equation from $\lambda =1$ to $\lambda =0$.

Bifurcation diagrams for the network equilibria can be found using a similar method. Let $\lambda$ be a network parameter of interest. If $(\textbf{H}_0,\textbf{P}_0,\lambda _0)$ is a solution to:

31 $$\begin{aligned} \textbf{F}(\textbf{H},\textbf{P},\lambda )={\textbf {0}}, \end{aligned}$$

then the bifurcation diagram of the equilibria of the system as $\lambda$ varies can also be found by tracking a solution to Eq. (31) using a similar Davidenko differential equation.

Further details on numerical continuation and our choice of the starting system $\textbf{G}$ can be found in Supplementary Material S2. We use Auto (Doedel et al 1999) to generate the bifurcation diagrams in Sect. 4.

## Results

In this section we investigate the relationship between network equilibria and flow in the redundant vessels. We begin by showing how the equilibria of the two networks in Fig. 2 change as we vary the length ratios, $\alpha$ and $\beta$, defined in Sect. 3.2.2. In Sect. 4.1 we vary $\alpha$ and $\beta$ to investigate the impact of the different length ratios on the number of equilibria. In Sect. 4.2 we investigate how the number of equilibria change as $\overline{H}_{in}$ and *D* vary. In Sect. 4.3 we vary the inlet pressures of the extended-triangle network, and show that the mechanism by which much equilibria emerge is independent of the choice of splitting rule.

### Effect of Varying Vessel Length Ratios on Network Equilibria

#### Vessel Length Asymmetry

In this section, we fix the length ratio of the redundant vessels ($\alpha = 0.1$), and investigate how the number and nature of the steady-state solutions (network equilibria) change as the length ratio associated with network asymmetry, $\beta$, is varied in the triangle and extended-triangle networks. We do not vary the reference length, $L$, because, as discussed in Sect. 3.2.1, it does not affect the normalised variables of the network and, hence, the qualitative behaviour of the model solutions.

**Fig. 3:**
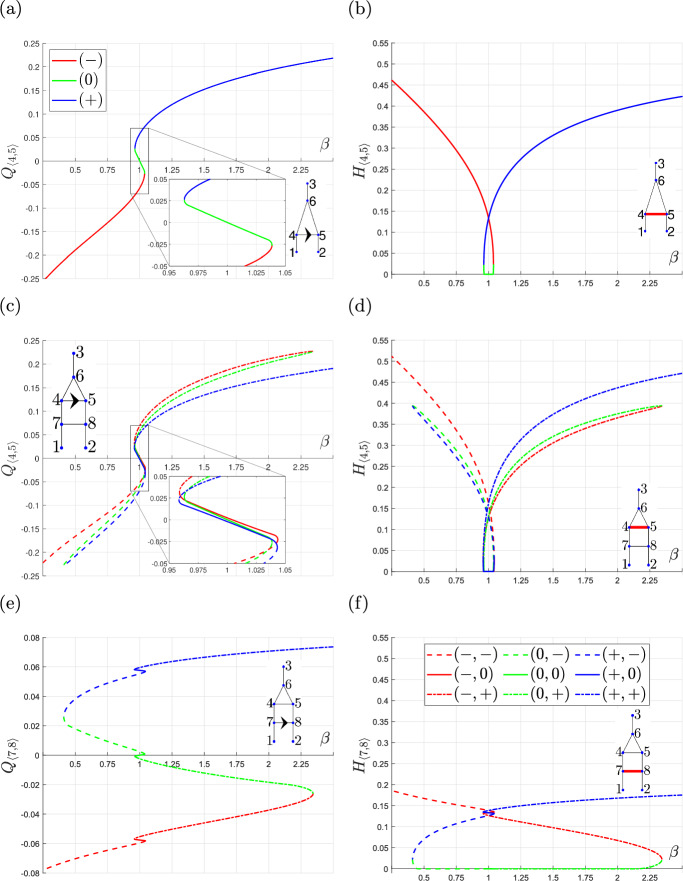
Series of bifurcation diagrams showing how the flow and haematocrit in the redundant vessels in the triangle and extended-triangle networks change as the length ratio $\beta$ varies. **a**, **b** volumetric flow $Q_{\langle 4,5 \rangle }$ and haematocrit $H_{\langle 4,5 \rangle }$ in the triangle network. **c**, **f** volumetric flows $Q_{\langle 7,8 \rangle }$ and $Q_{\langle 4,5 \rangle }$, and haematocrits $H_{\langle 7,8 \rangle }$ and $H_{\langle 4,5 \rangle }$, in the extended-triangle network. Key: each equilibrium is labelled $-,0$ or $+$ depending on whether the flow in the redundant vessels is negative, intermediate, or positive respectfully. The arrows on the network diagrams indicate the vessels for the corresponding flow plot, and defines which flow direction is considered positive

The results presented in Fig. 3a and b show how the equilibria of the triangle network change as $\beta$ varies. When $0 \le \beta \lesssim 0.96$, there is a unique solution for which $Q_{\langle 4,5 \rangle } < 0$. When $\beta \approx 0.96$, two additional solution branches emerge from a fold bifurcation, with $Q_{\langle 4,5 \rangle } > 0$ on both branches. As $\beta$ increases, one solution branch crosses $Q_{\langle 4,5 \rangle }=0$ and then collides with the solution branch for which $Q_{\langle 4,5 \rangle } < 0$ at a second fold bifurcation, when $\beta \approx 1.04$. The value of $Q_{\langle 4,5 \rangle }$ on this branch connecting the two fold bifurcations are intermediate between the corresponding values on the other two branches, for fixed values of $\beta$. The other solution branch persists for all values of $\beta \gtrsim 0.96$, and is characterised by $Q_{\langle 4,5 \rangle } > 0$. Therefore, the characteristics of the different solution branches are identifiable by their position on the S-shaped structure created by the two fold bifurcations. Taken together, Fig. 3a and b show that the solution branches can be distinguished by the flow in the redundant vessel, but that they are not uniquely defined by flow direction.

The flow pattern for equilibria on the solution branch connecting the two fold bifurcations motivates the following definition:

##### Definition 2

Let $\langle x,y \rangle$ be a redundant vessel in a network. Suppose that the network admits three equilibria which we denote *a*, *b* and *c*. Let $Q_{\langle x,y \rangle }^{(i)}, (i=$ a, b, c) denote the associated volumetric flow rates in the redundant vessel. Suppose, further, that equilibria *a* and *c* have negative and positive flows respectively, so that:

$$\begin{aligned} Q_{\langle x,y \rangle }^{{({a})}}< 0 < Q_{\langle x,y \rangle }^{{({c})}}. \end{aligned}$$

Equilibrium *b* is said to have *intermediate flow* in redundant vessel $\langle x,y\rangle$ if:

$$\begin{aligned} Q_{\langle x,y \rangle }^{{({a})}}< Q_{\langle x,y \rangle }^{{({b})}} < Q_{\langle x,y \rangle }^{{({c})}}. \end{aligned}$$

If the flow in the redundant vessel for a particular equilibrium does not satisfy the definition of intermediate flow, then the flow in the redundant vessel is termed positive or negative depending on its sign. Henceforth, we describe equilibria as having negative, intermediate or positive flow, or flow state, in a redundant vessel.

Applying Definition 2 to the triangle network (see Fig. 3a and b), we deduce that the solution branches are characterised by the type of flow in the redundant vessel:

$$\begin{aligned} (+) \;\;&\text{ for } \text{ positive } \text{ flow } \text{(blue } \text{ curve) }, \\ (0) \;\;&\text{ for } \text{ intermediate } \text{ flow } \text{(green } \text{ curve) }, \\ (-) \;\;&\text{ for } \text{ negative } \text{ flow } \text{(red } \text{ curve) }. \end{aligned}$$

For an equilibrium with intermediate flow, the haematocrit value in the redundant vessel is approximately 0 due to the form of the splitting rule. The flow ratio must exceed the threshold coefficient $$X_0$$ to realise a non-zero haematocrit value (see Eq. 13). For the triangle network, the equilibrium solution with intermediate flow has $$Q_{\langle 4,5 \rangle }=0$$ when $$\beta =1$$. We conclude that, when $$\beta \approx 1$$, the flow ratio is less than $$X_0 $$ and, consequently, there will be no haematocrit in the redundant vessel for most equilibria with intermediate flow (i.e., $$H_{\langle 4,5 \rangle } =0$$).

The asymmetric haematocrit distribution in vessels (4, 6) and (5, 6) is an emergent property of multiple equilibria in the triangle network. Figure S3 shows how $H_{(4,6)}$ and $H_{(5,6)}$ change as $\beta$ varies. For the $(+)$ equilibrium, we have

32 $$\begin{aligned} H_{(4,6)}> \overline{H}_{in}=0.45 > H_{(5,6)}, \end{aligned}$$

and conversely for the $(-)$ equilibrium. Inequality (32) holds when the triangle network is symmetric ($\beta =1$). Asymmetric haematocrit distributions in a symmetric triangle network arise when there is non-zero flow in the redundant vessel and due to the plasma skimming property of blood at bifurcation and convergence units (see Section S3 for details).

The notation for the flow state in a redundant vessel generalises naturally to networks with multiple redundant vessels. For example, since the extended-triangle network has two redundant vessels, the possible equilibria can be represented as follows:

$$\begin{aligned} (-,-),(-&,0),(-,+),\\ (0,-),(0&,0),(0,+),\\ (+,-),(+&,0), (+,+). \end{aligned}$$

As for the triangle network, the extended-triangle network admits multiple equilibria in a neighbourhood of $\beta =1$ (see Fig. 3c–f). When $\beta$ is sufficiently small or large, the extended-triangle network admits a unique equilibrium for which the flow direction in both redundant vessels is the same. We note that the extended-triangle network admits multiple equilibria for a larger range of values of $\beta$ than the triangle network (17.5 times larger).

A similar relationship between the flow in the redundant vessels and the equilibria can be observed in the bifurcation diagrams of the extended-triangle network. The bifurcation diagrams for the extended-triangle networks contain eight fold bifurcations, which we refer to as either inner or outer fold bifurcations, depending on their proximity to $\beta =1$. The two fold bifurcations at $\beta \approx 0.35$ and $\beta \approx 2.35$ are the two outer fold bifurcations, and the six fold bifurcations at $\beta \approx 0.96$ and $\beta \approx 1.04$ are the inner fold bifurcations. The bifurcation structure between the two outer fold bifurcations in Fig. 3e are similar to the S-shaped fold bifurcation structure for the triangle network in Fig. 3a. Applying the definition of intermediate flow to the solution branch connecting the two outer fold bifurcations reveals that these equilibria have intermediate flow in vessel $\langle 7,8 \rangle$. The equilibria belonging to the other two solution branches have either positive or negative flow depending on the sign of $Q_{\langle 7,8 \rangle }$. Each one of these solution branches contain two inner fold bifurcations, these two inner fold bifurcations also form an S-shaped fold bifurcation structure similar to that of the triangle network (See Fig. 3c). The solution branches connecting these inner fold bifurcations are characterised by intermediate flow in vessel $\langle 4,5 \rangle$, and the other two solution branches have either positive or negative flow in vessel $\langle 4,5 \rangle$. Therefore, all equilibria can be uniquely defined by the combination of flow in the two redundant vessels.

As with the triangle network, for a given equilibrium solution, haematocrit values in the redundant vessels can be used to identify redundant vessels with intermediate flow: $H_{\langle 7,8 \rangle } \approx 0$ for the $(0,-),(0,0)$ and $(0,+)$ equilibria, and $H_{\langle 4,5 \rangle } \approx 0$ for the $(-,0),(0,0)$ and $(+,0)$ equilibria. The haematocrit in the redundant vessels is approximately 0 for the same reason that the haematocrit in the redundant vessel of the triangle network was approximately 0 for the equilibria with intermediate flow (Fig. 3b).

The overlap of the regions generated by the inner S-shaped bifurcation structures on the three coloured solution branches creates a range of values of $\beta$ for which 9 equilibria exist; and there is a distinct equilibrium for each combination of flow states in the redundant vessels. This interval is similar in size to that for which the triangle network admits multiple equilibria. We note further that the range of $\beta$ values for which the extended-triangle network admits multiple equilibria can be subdivided into smaller sub-intervals containing 3, 5, 7 and 9 equilibria.

Every equilibrium of the two networks has a unique combination of flow states in the two redundant vessels. Therefore, the maximum number of equilibria admitted by the triangle and extended-triangle networks are 3 and 9 respectively. However, the networks do not always admit the maximum number of equilibria. Indeed, not every flow configuration is viable for all sets of parameter values. Regardless of whether a network admits the maximum number of equilibria, Fig. 3 illustrates that equilibria can be distinguished by the type of flow in at least one redundant vessel.

#### Effect of Varying the Length of the Redundant Vessels

**Fig. 4:**
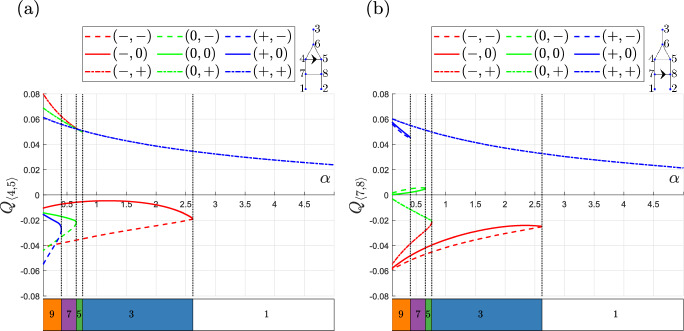
Bifurcation diagrams showing how the equilibria of the extended-triangle network change as $\alpha$ varies when $\beta = 1.025$. **a** $Q_{\langle 4,5 \rangle }$ . **b** $Q_{\langle 7,8 \rangle }$. Key: for each equilibrium, the flow in the redundant vessels is labelled $-,0$ or $+$ if it is negative, intermediate, or positive, respectfully, depending on the flow state when $\alpha =0.1$. As $\alpha$ increases, the equilibria meet at fold bifurcations beyond which the equilibria cease to be viable

Fig. 3 demonstrates that the extended-triangle network possesses more equilibria than the triangle network and that it admits multiple equilibria for a wider range of values of $\beta$ when we fix $\alpha = 0.1$. Recall that $\alpha$ is the ratio of the length of the redundant vessels to the reference vessel length, and that the hydraulic resistance in a vessel is proportional to its length. Figure 4 shows how the number of equilibria in the extended-triangle network changes when we vary $\alpha$ and fix $\beta = 1.025$. As $\alpha$ increases, the resistance to flow in the redundant vessels increases. When the resistance becomes sufficiently large, certain equilibria cease to be valid and pairs of solutions meet at fold bifurcations, causing the number of equilibria to decrease from 9 to 1 as $\alpha$ increases.

#### Effect of Varying $\alpha$ and $\beta$

**Fig. 5:**
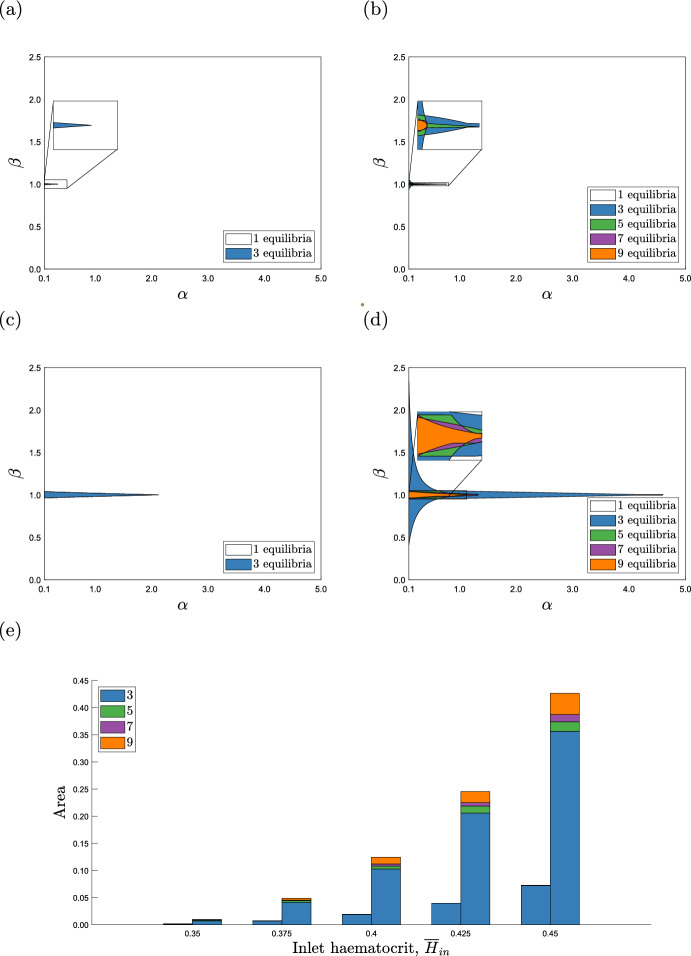
Regions distinguished by the number of equilibria admitted by the triangle and extended-triangle networks, as the length ratios $\alpha$ and $\beta$ vary for different inlet haematocrit values, $\overline{H}_{in}$. **a**, **b** The regions of $(\alpha , \beta )$ parameter space for the triangle and extended-triangle networks, when $\overline{H}_{in} = 0.35$, respectively. **c**, **d** The regions of $(\alpha , \beta )$ parameter space for the triangle and extended-triangle networks, when $\overline{H}_{in} = 0.45$, respectively. **e** Stacked bar chart showing how the size of the areas of ($\alpha , \beta$) parameter space in which the two networks admit multiple equilibria change as we vary the inlet haematocrit, $\overline{H}_{in}$. The left bar stack of each pair corresponds to the triangle network and the right stack to the extended-triangle network. The insets in (**a**), (**b**) and (**d**) are zoomed in views of the region surrounding $\beta =1$. As expected, the extended-triangle network admits multiple equilibria over a larger region of $(\alpha , \beta )$ parameter space

Figs. 3 and 4 show how the bifurcation structures of the triangle and extended-triangle networks change as the length ratios $\beta$ and $\alpha$ vary. We now perform a more comprehensive study, to investigate how the number of equilibria change as $\alpha$ and $\beta$ vary, for fixed values of the inlet haematocrit, $\overline{H}_{in}$. The results presented in Fig. 5a and c show that, when $\overline{H}_{in}=0.35$ and $\overline{H}_{in}=0.45$ respectively, the triangle network admits a unique equilibrium for most values of $\alpha$ and $\beta$ (we note that Fig. 5c shows the same information about the triangle network as Fig. 5c and d in (Gardner et al 2010)).There is a small, tapering region where $|\beta -1|$ and $\alpha$ are small, in which three equilibria exist but this region is much larger when $\overline{H}_{in}=0.45$. Figure 5b and d reveal a similar, but more detailed, structure for the extended-triangle network, when $\overline{H}_{in}=0.35$ and $\overline{H}_{in}=0.45$ respectively. There is a small, tapering region where $|\beta - 1|$ and $\alpha$ are small, in which the extended-triangle network admits 9 equilibria. This region is surrounded by narrow regions in which the network admits 7, 5 and 3 equilibria. Outside this region of $(\alpha , \beta )$ parameter space, the extended-triangle network admits a unique equilibrium. Figure 5e shows how the area of the region of ($\alpha , \beta$) parameter space in which the models exhibit multiple equilibria changes as the inlet haematocrit varies. For both networks, the total area increases as the inlet haematocrit increases, revealing that multiple equilibria are more prevalent in the $(\alpha , \beta )$ plane as $\overline{H}_{in}$ increases.

As in Fig. 3, all equilibria have unique flow configurations. When $\beta =1$ and the networks are symmetric, blood may flow in either direction through the redundant vessels, and so the networks admit more flow configurations when $|\beta - 1| \ll 1$. When $|\beta -1| \gg 1$, the degree of network asymmetry increases and, for most values of $\alpha$, both networks admit a unique equilibrium. If, however, $\alpha$ is sufficiently small, then we continue to observe multiple equilibria: the shorter redundant vessels compensate for the increased network asymmetry by offering less resistance to blood flow. The extended-triangle network can accommodate a greater degree of asymmetry because it has two redundant vessels in which the blood flows in parallel, thus, the resistance is reduced compared to the single redundant vessel in the triangle network. This effect is significant because the interval of $\beta$ for which the extended-triangle network admits multiple equilibria is up to 17.5 times larger than the interval for the triangle network when $\overline{H}_{in} = 0.45$ (see Fig. 5c and d).

### Effect of Varying Vessel Diameters and Inlet Haematocrit on the Number of Equilibria of the Extended-Triangle Network

In Sect. 4.1 we observed that hydraulic resistance influences the number of equilibria in a given network. In this section we extend that analysis by studying the effect of resistance changes induced by varying the blood viscosity. As discussed in Sect. 3.2.1, the relative viscosity of the blood depends nonlinearly on the vessel’s haematocrit and diameter values (Eq. 3). When $D$ is varied, this nonlinearity alters the daughter to parent vessel flow ratios at equilibrium and, hence, the haematocrit values also change. Accordingly, we now investigate the effect of varying the vessel diameter $D$ and the inlet haematocrit $\overline{H}_{in}$.

**Fig. 6:**
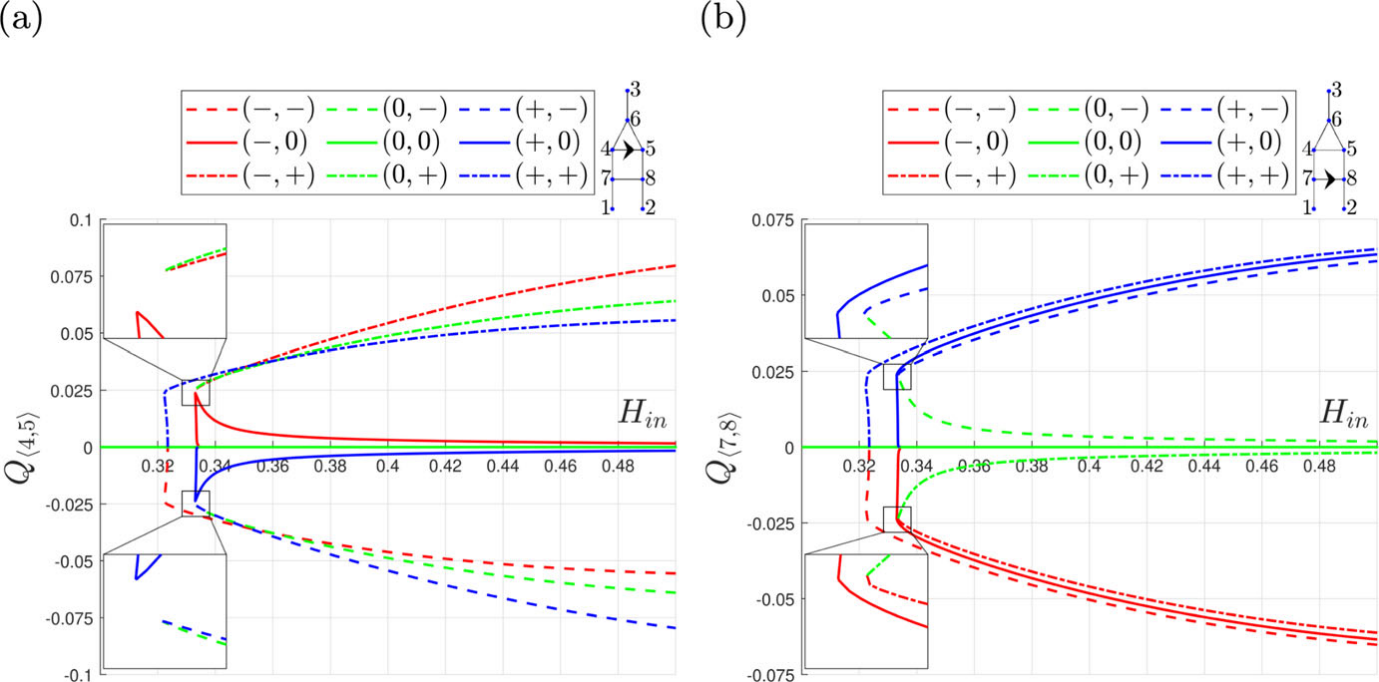
Bifurcation analysis of the equilibria of a symmetric extended-triangle network (Fig. 2b, with $\beta =1$, $\alpha = 0.1$) as the inlet haematocrit varies. We plot the flow in the redundant vessels. **a** $Q_{\langle 4,5 \rangle }$, **b** $Q_{\langle 7,8 \rangle }$) associated with each equilibrium as the inlet haematocrit varies. Solution branches associated with different flow configurations meet at fold bifurcations whose locations determine the smallest haematocrit values $\overline{H}_{in}^{(3)}, \overline{H}_{in}^{(5)},$ and $\overline{H}_{in}^{(9)}$, at which the network admits 3, 5 and 9 equilibria, respectively. The insets are magnify the behaviour near points of interest

In Fig. 6 we show how the equilibria of the extended-triangle network change when $D= 10$, and $\overline{H}_{in}$ varies. When $\overline{H}_{in} = 0$, there is no haematocrit in any vessel and the network equations are linear, and so the extended-triangle network admits a unique equilibrium for which $Q_{\langle 7,8 \rangle } = Q_{\langle 4,5 \rangle } =0$. As $\overline{H}_{in}$ increases, equilibria emerge at fold bifurcations until 9 equilibria with unique flow configurations exist. We term the values of $\overline{H}_{in}$ at these fold bifurcations as critical values of the extended-triangle network. The value of $\overline{H}_{in}$ at the fold bifurcations close to $\overline{H}_{in} \approx 0.32$ coincides with the emergence of $(+,+)$ and $(-,-)$ equilibria and, therefore, we refer to this critical value as $\overline{H}_{in}^{(3)}$. The extended-triangle network admits 5 equilibria in the interval $(\overline{H}_{in}^{(3)}, \overline{H}_{in}^{(3)}+\epsilon )$. However, since $0 < \epsilon \ll 1$, and two of the equilibria meet at a bifurcation, we neglect this small interval of 5 equilibria when describing the bifurcation structure. We also identify $\overline{H}_{in}^{(5)}$ and $\overline{H}_{in}^{(9)}$, critical values of $\overline{H}_{in}$ at which 5 and 9 equilibria emerge, respectively.

**Fig. 7:**
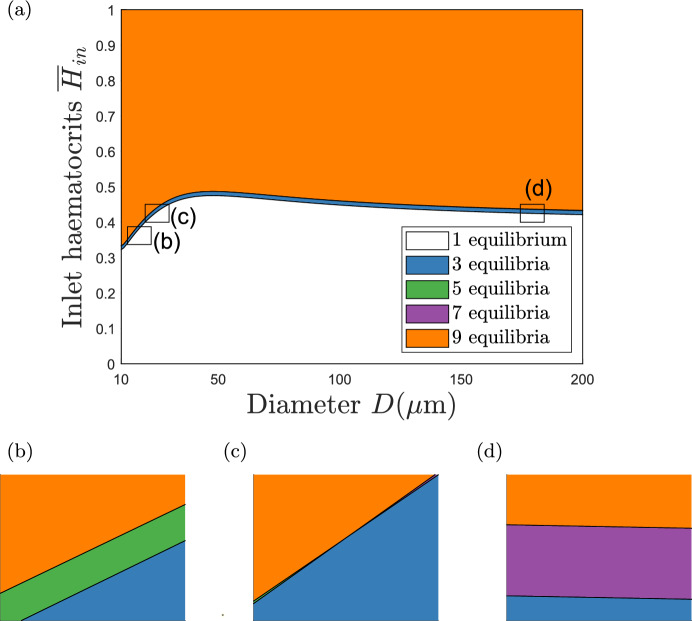
Bifurcation diagram showing how the $(D,\overline{H}_{in})$ plane partitions into distinct regions according to the number of equilibria admitted by the extended-triangle network

We identified the critical inlet haematocrit values for $D\in [10,200]$. Figure 7 partitions the $(D,\overline{H}_{in})$ plane into distinct regions according to the number of equilibria admitted by the extended-triangle network. The boundary between these regions are critical values of the inlet haematocrits. As in Fig. 6, the extended-triangle network admits a unique equilibrium when $\overline{H}_{in} = 0$, and the number of equilibria increases as $\overline{H}_{in}$ increases until the network admits 9 equilibria. This trend is consistent for all values of $D$, and shows that the network is more likely to admit multiple equilibria when the value of $\overline{H}_{in}$ is large. One possible explanation for this phenomenon is that multiple flow pathways arise in response to more RBCs in the network. When the inlet haematocrit is small, the RBCs have sufficient space within the side vessels, and the redundant vessels remain empty or almost empty. Larger inlet haematocrit values introduce additional RBCs that are redirected down the redundant vessels to account for the increased mass moving through the network. As the extended-triangle network is geometrically symmetric, the blood flowing in one direction in a redundant vessel is equivalent to the blood flowing in the opposite direction, therefore, the number of equilibria increases due to the increased number of viable flow pathways.

In Fig. 6, the values of $\overline{H}_{in}^{(3)}$ and $\overline{H}_{in}^{(9)}$ are close together when $D=10$. This behaviour is mirrored by the small region of 3 equilibria that separates regions of 1 and 9 equilibria in Fig. 7. The insets in Fig. 6 also reveal a region of 5 equilibria which emerges at a critical value of $\overline{H}_{in}^{(5)}$ for which $(+,0)$ and $(-,0)$ equilibria are created. This region is small in comparison to the size of the other regions because the critical value $\overline{H}_{in}^{(9)}$, at which the $(+,-),(0,-),(0,+)$ and $(-,+)$ equilibria emerge, is only slightly larger than $\overline{H}_{in}^{(5)}$ ($0 < \overline{H}_{in}^{(9)} - \overline{H}_{in}^{(5)} \ll 1$). This trend continues as $D$ increases until $D\approx 25$. At this vessel diameter, the network admits a critical value $\overline{H}_{in}^{(7)}$ instead of $\overline{H}_{in}^{(5)}$ because, as $\overline{H}_{in}$ increases, the $(+,-),(0,-),(0,+)$ and $(-,+)$ equilibria emerge before the $(+,0)$ and $(-,0)$ equilibria. This new region of 7 equilibria remains small. The regions containing 5 and 7 equilibria are visible in Fig. 7b and d, and the point at which the network transitions between the two regions is visible in Fig. 7c.

In Fig. 7, the curves separating regions of the $(D,\overline{H}_{in})$ plane containing 1, 3 and 9 equilibria are approximately parallel to each other. On these critical curves, $\overline{H}_{in}^{(3)}$ and $\overline{H}_{in}^{(9)}$ attain their maximum values at approximately the same value of $D$ ($D\approx 50$), and then decrease to constant values as $D\rightarrow \infty$. A similar pattern was observed by Gardner et al (2010) for the triangle network when the same parameters were varied (see Figure 7a in their paper).

The functional form of the relative viscosity provides a heuristic explanation of this behaviour. Recall that

33 $$\begin{aligned} \mu (H,D) = \mu _p \Bigg [1 + (\mu _{45} - 1) \bigg ( \frac{(1 - H)^C - 1}{0.55^C - 1} \bigg ) \bigg (\frac{D}{D- 1.1}\bigg )^2 \Bigg ] \bigg (\frac{D}{D- 1.1}\bigg )^2.\nonumber \\ \end{aligned}$$

If $D \gg 10$, then, from Eqs. (5 and 4) the parameters *C* and $\mu _{45}$ are approximately constant:

34 $$\begin{aligned} C \approx -0.8 \quad \text{ and } \quad \mu _{45} \approx 3.2, \end{aligned}$$

and Eq. (33) reduces to give

35 $$\begin{aligned} \mu (H,D) \approx \mu _p \bigg ( 1 + 2.2 \bigg ( \frac{(1 - H)^{-0.8} - 1}{0.55^{-0.8} - 1} \bigg )\mu _p \equiv \mu _{\infty }^{(rel)}(H). \end{aligned}$$

With $\mu ^{(rel)}(H,D) \approx \mu _{\infty }^{(rel)}(H)$, the volumetric flow $Q_{(x,y)}$ in vessel (*x*, *y*) can be approximated as follows:

36 $$\begin{aligned} Q_{(x,y)} \approx \frac{(P_x-P_y) D^4 \pi }{128 L_{(x,y)} \mu _p \mu _{\infty }^{(rel)}(H_{(x,y)})}. \end{aligned}$$

As the network equations are defined in terms of flow ratios, if we use Eq. (36) to approximate Eq. (9) and scale by the inlet flow (see Eq.  (25)), then the network equilibria do not change as *D* varies. We conclude that for sufficiently large values of *D* ($D\gg 10$), the network equilibria are independent of the diameter *D*.

The above explanation neglects the impact that vessel diameters have on the splitting rules. In practice, the coefficients of the splitting rule usually depend on vessel diameters. Consider, for example, the model proposed by Pries et al (1990), where the splitting rule depends on the vessel diameters (Eqs. (14–16)). However, these coefficients can also be approximated by constants for sufficiently large values of $D$, therefore, the effect of the coefficients of the splitting rule are also negligible.

Although we have not considered vessel length in this section, we note that the vessel length to diameter ratio might be unrealistic for a range of values of $D$ that we have considered. However, we have chosen not to vary the reference vessel length $L$ because as discussed in Sects. 3.2.1 and 4.1.1, the value of $L$ does not affect the normalised variables.

### Different Splitting Rules

In addition to varying geometric parameters for the triangle and extended-triangle networks, we also varied the inlet pressures (Figures S4 and S5) for the extended-triangle network and compared the equilibrium solutions for five splitting rules, including the Pries 1990 splitting rule. Our results are presented in Supplementary Material S4 and show that the equilibria emerge in sets of three, distinguished by the flow in one of the redundant vessels, regardless of the choice of splitting rule.

## Conclusions

In this paper, we have performed a systematic bifurcation analysis of two idealised networks, the triangle and extended-triangle networks, to investigate how the number of steady flow solutions changes as vessel length ratios and vessel diameters vary. In so doing, we have identified a consistent relationship between multiple equilibria and network redundancy, whereby multiple equilibria arise from the multiple flow directions that can be realised in redundant vessels. Other authors have identified multiple equilibria in idealised networks and observed that some of these equilibria differ in terms of the flow direction through one or more vessels (Gardner et al 2010; Karst et al 2017). However, to our knowledge, we are the first to identify redundant vessels as the key vessels for generating multiple equilibria. Furthermore, we can distinguish equilibria by characterising the flow in the redundant vessels as negative, intermediate or positive. By focusing on the triangle and extended-triangle networks, we have demonstrated the impact of additional network redundancy on the multiplicity of equilibria. By extrapolating our findings, we predict that the number of equilibria that a network admits is limited by the number of permutations of negative, intermediate and positive flow across its redundant vessels.

The type of flow in the redundant vessels is identifiable from the structure of the bifurcation diagrams. For example, the solution branches of the bifurcation diagrams of the triangle and extended-triangle networks in Fig. 3 contain a common S-shaped bifurcation structure coinciding the with different flow states in the redundant vessels. Both networks have a common length ratio, $\beta$, which controls the degree of network asymmetry. The bifurcation diagram of the flow in the redundant vessel of the triangle network in Fig. 3a contains the S-shaped structure created by the two fold bifurcations as $\beta$ varies. Therefore, the equilibria belonging to the solution branch connecting the two fold bifurcations clearly have intermediate flow.

The bifurcation diagram for the extended-triangle network as $\beta$ varies in Fig. 3c and e shares the same S-shaped fold bifurcation structure. We distinguish between fold bifurcations base upon their distance to $\beta =1$: we refer to fold bifurcations close to this value as inner fold bifurcations, and bifurcations which are further away are referred to as outer fold bifurcations. The bifurcation diagram contained two outer fold bifurcations, and six inner fold bifurcations. The structure of the solution branches between the outer fold bifurcations is similar to the S-shaped bifurcation structure observed in the bifurcation diagram for the triangle network. Therefore, the extended-triangle network admits three solution branches between the two outer fold bifurcations which are characterised by different flow in the first redundant vessel, closest to the two inlets. Each one of these solution branches contain a S-shaped bifurcation structure coinciding with different flow states in the second redundant vessel. The S-shaped bifurcation structure, for each of the redundant vessels, was also present when the inlet pressures of the extended-triangle network were varied in Sect. 4.3. Hence the bifurcation diagrams of varying $\beta$ and the inlet pressures could be used to identify the combinations of flow in the two redundant vessels of the extended-triangle network.

Network redundancy does not guarantee the existence of multiple equilibria. For example, as a network becomes more asymmetric, the number of equilibria decreases, with highly asymmetric networks admitting a unique equilibrium. While it is not feasible to exhaustively test every parameter in the two networks studied in this paper, the relationship between the existence of multiple equilibria and flow is consistent across a wide range of vessel length ratios and vessel diameters. Furthermore, network redundancy may increase the range of parameter values for which multiple equilibria are found. For example, the interval of $\beta$ for which the extended-triangle network admitted multiple equilibria was 17.5 times larger than in the interval of multiple equilibria for the triangle network in Fig. 3. This effect is particularly prominent when the redundant vessels are short relative to other vessels (see Fig. 5). Building upon this result, we hypothesise that networks with shorter redundant vessels, a characteristic of vasculature known to be present in tumours (Bernabeu et al 2020), are more likely to admit multiple equilibria.

Our results suggest that networks which are symmetric and contain more redundant vessels admit more equilibria than asymmetric networks with fewer redundant vessels. However, in diseases, such as cancer, the vasculature is often highly irregular, with heterogeneity in vascular density, and abnormal dilation in some vessels (Jain 2005). Our network analysis suggests that such asymmetric tumour vasculature is unlikely to admit multiple equilibria. However, our results also indicate that networks with a greater number of redundant vessels admit multiple equilibria across a wider range of parameter values. Therefore, if a tumour vascular network contains greater redundancy than the surrounding healthy vasculature, then we predict that it will admit more equilibria than the healthy vasculature. In order to estimate a network’s redundancy, we can use Topological Data Analysis (TDA) to count the number of looped structures. Consider, for example, the difference between the triangle and extended triangle networks: the extended triangle network contains an additional loop and an additional redundant vessel. Stolz et al (2022) used TDA to quantify the number of loops in large, tumour vascular networks. Their analysis revealed that networks exposed to anti-angiogenic agents contained fewer loops than untreated networks. In future work, it would be interesting to investigate the relationship between the number of equilibria and the number of loops admitted by a tumour vascular network.

Most studies linking blood flow to the emergence of hypoxia in tumours conclude that hypoxia arises from the non-uniform distribution of haematocrit associated with a unique stable equilibrium (Bernabeu et al 2020; Sweeney et al 2018). The source of heterogeneity is often attributed to abnormal vessel morphology in large heterogeneous vascular networks. Here, we have shown that relatively small networks with redundant vessels may admit multiple equilibria. Furthermore, in Sect. 4.1, we saw how network redundancy can generate flow solutions with asymmetric haematocrit distributions in the symmetric triangle network. We conclude that multiple equilibria could be an additional cause of hypoxia within tumours. Therefore, in future work it would be of interest to investigate whether the existence of multiple equilibria is linked with heterogeneous haematocrit distributions in large vascular networks.

It would also be of interest to characterise the stability of the different equilibria and to investigate whether the networks admit oscillatory solutions. Ben-Ami et al (2022) linked multiple equilibria with the existence of oscillatory blood flow dynamics, and suggested that similar oscillations may be responsible for cycling hypoxia in the tumour microenvironment (ie short term fluctuations between normoxic and hypoxic conditions(Michiels et al 2016)). Another possible cause of cycling hypoxia, that we will investigate in future work, is stochastic switching between different stable equilibria.

## Supplementary Information

Below is the link to the electronic supplementary material.

Supplementary file 1 (pdf 965 KB)
