# Supplementary material for: Key Structural Features of Microvascular Networks Leading to the Formation of Multiple Equilibria
Source: Bull Math Biol. 2025 Jan 23;87(2):30. doi: 10.1007/s11538-024-01404-y (PMC11757897; doi:10.1007/s11538-024-01404-y)
Supplement: Supplementary file 1 — (pdf 965 KB) [file 11538_2024_1404_MOESM1_ESM.pdf]

S1 Network equations

All network equations can be derived from the network geometry and the equation construction in Section 3.1. This section contains the network equations for all networks studied in this publication.

S1.1 Triangle network

The geometry of the triangle network can be seen in Figure 2a. This network requires three pressure boundary conditions: $\overline{P}_1, \overline{P}_2$ and $\overline{P}_3$; and two haematocrit boundary conditions: $\overline{H}_{(1,4)}$ and $\overline{H}_{(2,5)}$. The steady-state equations for the triangle network are as follows:

$$0 = Q_{(1,4)} + Q_{(6,4)} + Q_{\langle 5,4\rangle}, \tag{S1}$$

$$0 = Q_{(2,5)} + Q_{(6,5)} + Q_{\langle 4,5\rangle}, \tag{S2}$$

$$0 = Q_{(3,6)} + Q_{(4,6)} + Q_{(5,6)}, \tag{S3}$$

$$H_{(4,6)} = \begin{cases} \frac{\psi_{(4,6)}(Q_{(4,6)}/Q_{(1,4)},\overline{H}_{(1,4)})\overline{H}_{(1,4)}Q_{(1,4)}}{Q_{(4,6)}} & Q_{\langle 4,5\rangle} > 0, \\ \frac{\overline{H}_{(1,4)}Q_{(1,4)}+H_{\langle 4,5\rangle}Q_{\langle 5,4\rangle}}{Q_{(4,6)}} & Q_{\langle 4,5\rangle} \leq 0, \end{cases} \tag{S4}$$

$$H_{(5,6)} = \begin{cases} \frac{\psi_{(5,6)}(Q_{(5,6)}/Q_{(2,5)},\overline{H}_{(2,5)})\overline{H}_{(2,5)}Q_{(2,5)}}{Q_{(5,6)}} & Q_{\langle 4,5\rangle} < 0, \\ \frac{\overline{H}_{(2,5)}Q_{(2,5)}+H_{\langle 4,5\rangle}Q_{\langle 4,5\rangle}}{Q_{(5,6)}} & Q_{\langle 4,5\rangle} \geq 0, \end{cases} \tag{S5}$$

$$H_{\langle 4,5\rangle} = \begin{cases} \frac{\psi_{\langle 5,4\rangle}(Q_{\langle 5,4\rangle}/Q_{(2,5)},\overline{H}_{(2,5)})\overline{H}_{(2,5)}Q_{(2,5)}}{Q_{\langle 5,4\rangle}} & Q_{\langle 4,5\rangle} < 0, \\ \frac{\psi_{\langle 4,5\rangle}(Q_{\langle 4,5\rangle}/Q_{(1,4)},\overline{H}_{(1,4)})\overline{H}_{(1,4)}Q_{(1,4)}}{Q_{\langle 4,5\rangle}} & Q_{\langle 4,5\rangle} > 0, \\ 0 & Q_{\langle 4,5\rangle} = 0, \end{cases} \tag{S6}$$

$$H_{(6,3)} = \frac{H_{(4,6)}Q_{(4,6)} + H_{(5,6)}Q_{(5,6)}}{Q_{(6,3)}}. \tag{S7}$$

S1.2 Square plus triangle network

The geometry of the square plus triangle network can be seen in Figure 2b. This network requires three pressure boundary conditions: $\overline{P}_1, \overline{P}_2$ and $\overline{P}_3$; and two haematocrit boundary conditions: $\overline{H}_{(1,7)}$ and $\overline{H}_{(2,8)}$. The steady-state equations for the square plus triangle network are as follows:

$$0 = Q_{(1,7)} + Q_{(4,7)} + Q_{\langle 8,7\rangle}, \tag{S8}$$

$$0 = Q_{(2,8)} + Q_{(5,8)} + Q_{\langle 7,8\rangle}, \tag{S9}$$

$$0 = Q_{(6,4)} + Q_{(7,4)} + Q_{\langle 5,4\rangle}, \tag{S10}$$

$$0 = Q_{(6,5)} + Q_{(8,5)} + Q_{\langle 4,5\rangle}, \tag{S11}$$

$$0 = Q_{(3,6)} + Q_{(4,6)} + Q_{(5,6)}, \tag{S12}$$

$$H_{(7,4)} = \begin{cases} \frac{\psi_{(7,4)}(Q_{(7,4)}/Q_{(1,7)},\overline{H}_{(1,7)})\overline{H}_{(1,7)}Q_{(1,7)}}{Q_{(7,4)}} & Q_{\langle 7,8\rangle} > 0, \\ \frac{\overline{H}_{(1,7)}Q_{(1,7)}+H_{\langle 7,8\rangle}Q_{\langle 8,7\rangle}}{Q_{(7,4)}} & Q_{\langle 4,5\rangle} \leq 0, \end{cases} \tag{S13}$$

$$H_{(8,5)} = \begin{cases} \frac{\psi_{(8,5)}(Q_{(8,5)}/Q_{(2,8)},\overline{H}_{(2,8)})\overline{H}_{(2,8)}Q_{(2,8)}}{Q_{(8,5)}} & Q_{\langle 7,8\rangle} < 0, \\ \frac{\overline{H}_{(2,8)}Q_{(2,8)}+H_{\langle 7,8\rangle}Q_{\langle 7,8\rangle}}{Q_{(8,5)}} & Q_{\langle 7,8\rangle} \geq 0, \end{cases} \tag{S14}$$

$$H_{\langle 7,8\rangle} = \begin{cases} \frac{\psi_{\langle 8,7\rangle}(Q_{\langle 8,7\rangle}/Q_{(2,8)},\overline{H}_{(2,8)})\overline{H}_{(2,8)}Q_{(2,8)}}{Q_{\langle 8,7\rangle}} & Q_{\langle 7,8\rangle} \leq 0, \\ \frac{\psi_{\langle 7,8\rangle}(Q_{\langle 7,8\rangle}/Q_{(1,7)},\overline{H}_{(1,7)})\overline{H}_{(1,7)}Q_{(1,7)}}{Q_{\langle 7,8\rangle}} & Q_{\langle 7,8\rangle} > 0, \\ 0 & Q_{\langle 7,8\rangle} = 0, \end{cases} \tag{S15}$$

$$H_{(4,6)} = \begin{cases} \frac{\psi_{(4,6)}(Q_{(4,6)}/Q_{(7,4)},H_{(7,4)})H_{(7,4)}Q_{(7,4)}}{Q_{(4,6)}} & Q_{\langle 5,4\rangle} > 0, \\ \frac{H_{(7,4)}Q_{(7,4)}+H_{\langle 4,5\rangle}Q_{\langle 5,4\rangle}}{Q_{(4,6)}} & Q_{\langle 4,5\rangle} \leq 0, \end{cases} \tag{S16}$$

$$H_{(5,6)} = \begin{cases} \frac{\psi_{(5,6)}(Q_{(5,6)}/Q_{(8,5)},H_{(8,5)})H_{(8,5)}Q_{(8,5)}}{Q_{(5,6)}} & Q_{\langle 4,5\rangle} < 0, \\ \frac{H_{(8,5)}Q_{(8,5)}+H_{\langle 4,5\rangle}Q_{\langle 4,5\rangle}}{Q_{(5,6)}} & Q_{\langle 4,5\rangle} \geq 0, \end{cases} \tag{S17}$$

$$H_{\langle 4,5\rangle} = \begin{cases} \frac{\psi_{\langle 4,5\rangle}(Q_{\langle 4,5\rangle}/Q_{(7,4)},H_{(7,4)})H_{(7,4)}Q_{(7,4)}}{Q_{\langle 5,4\rangle}} & Q_{\langle 4,5\rangle} < 0, \\ \frac{\psi_{\langle 5,4\rangle}(Q_{\langle 5,4\rangle}/Q_{(8,5)},H_{(8,5)})H_{(8,5)}Q_{(8,5)}}{Q_{\langle 4,5\rangle}} & Q_{\langle 4,5\rangle} > 0, \\ 0 & Q_{\langle 4,5\rangle} = 0, \end{cases} \tag{S18}$$

$$H_{(6,3)} = \frac{H_{(4,6)}Q_{(4,6)} + H_{(5,6)}Q_{(5,6)}}{Q_{(6,3)}}. \tag{S19}$$

S2 Numerical continuation

In Section 3.3 we describe the use of numerical continuation to both find an initial equilibrium of the system of network equations described in Section 3.1, and then study their trajectory in parameter space as we vary different network parameters. In this supplementary information, we will provide more detail on how we use continuation to find solutions to the network equations, including our choice of starting system $\mathbf{G}(\mathbf{H}, \mathbf{P})$, and the process by which we find an initial solution to the network equations and then create a bifurcation diagram as a network parameter is varied. More details of these techniques may be found in books, e.g. (Allgower and Georg, 2003; Kuznetsov et al, 1998).

S2.1 Finding an initial equilibrium

The homotopy function for finding an initial equilibrium of the system of network equations takes the following form:

$$\mathbf{h}(\mathbf{H}, \mathbf{P}, \lambda) = \mathbf{F}(\mathbf{H}, \mathbf{P})(1 - \lambda) + \mathbf{G}(\mathbf{H}, \mathbf{P})\lambda, \tag{S20}$$

where $\mathbf{F} = \mathbf{0}$ is the system of network equations described in Section 3.1 and $\mathbf{G} = \mathbf{0}$ is a starting system. We considered two different choices for this starting system. The first choice of $\mathbf{G}$ is that of the network flow equations with a constant haematocrit distribution. In this case, fixing the haematocrit distribution uniquely defines the pressures in the network because the conservation of flow equations are linear for a fixed haematocrit distribution. For a network $\mathcal{N} = \{N, E\}$, the functions associated with the pressure and haematocrit variables for the starting system $\mathbf{G} = \mathbf{0}$ take the following forms:

$$G_v(\mathbf{H}, \mathbf{P}) = Q_{(u,v)} + Q_{(w,v)} + Q_{(z,v)}, \tag{S21}$$

$$G_{(v,w)}(\mathbf{H}, \mathbf{P}) = H_{(v,w)} - H^*_{(v,w)}, \tag{S22}$$

where G_v is the function associated with the pressure variable P_v, $G_{(v,w)}$ is the function associated with the haematocrit variable $H_{(v,w)}$, and $H^*_{(v,w)}$ is a constant haematocrit that has been set. This means that the solution to $\mathbf{G} = \mathbf{0}$ is specified by the chosen constant haematocrit distribution $\mathbf{H}^*$.

As the function $F_v(\mathbf{H}, \mathbf{P})$, associated with the pressure variable P_v, (Equation (18)) is the same as $G_v(\mathbf{H}, \mathbf{P})$:

$$G_v(\mathbf{H}, \mathbf{P}) = F_v(\mathbf{H}, \mathbf{P}) = h_v(\mathbf{H}, \mathbf{P}, \lambda). \tag{S23}$$

Regardless of whether $F_{(v,w)}$ takes the form of Equations (19) or (20), the homotopy function, $h_{(v,w)}$, associated with the haematocrit variable $H_{(v,w)}$, takes the following form:

$$h_{(v,w)}(\mathbf{H}, \mathbf{P}, \lambda) = F_{(v,w)}(\mathbf{H}, \mathbf{P})(1 - \lambda) + (H_{(v,w)} - H^*_{(v,w)})\lambda, \tag{S24}$$

where λ is a continuation parameter. The homotopy function is easily adaptable if a continuation is unsuccessful in obtaining an equilibrium of the network. A new constant haematocrit distribution, $\mathbf{H}^*$, is chosen until an equilibrium is obtained. Although in most cases this is sufficient to perform a successful continuation, in general, it is not possible to determine if a path between the solutions of the starting system and target system exists.

The second starting system we consider is that of the network equations with a different splitting rule. If the solutions to the network equations for the splitting rule ϕ are known, then the network equations with ϕ as the splitting rule can be used as the starting system. Let $\mathbf{F}^{(\psi)}(\mathbf{H}, \mathbf{P}) = \mathbf{0}$ denote the system of equations for a network using the splitting rule ψ and let $\mathbf{F}^{(\phi)}(\mathbf{H}, \mathbf{P}) = \mathbf{0}$ denote the system of equations for a network using the splitting rule ϕ. Then the homotopy function converting the solutions to the network equations using the ϕ splitting rule to the ψ splitting rule takes the following form:

$$\mathbf{h}(\mathbf{H}, \mathbf{P}, \lambda) = \mathbf{F}^{(\phi)}(\mathbf{H}, \mathbf{P})\lambda + \mathbf{F}^{(\psi)}(\mathbf{H}, \mathbf{P})(1 - \lambda). \tag{S25}$$

One strategy for finding solutions to the network equations for different splitting rules may be to start with a constant haematocrit distribution to find the solutions to the network equations for one splitting rule, then use these equilibria to find the equilibria and the network equations using other splitting rules.

S2.2 Tracking equilibria in parameter space

Once at least one equilibrium of a network is known, numerical continuation can be used to investigate how the equilibria change as certain parameters vary. For this purpose, the homotopy function is as follows:

$$\mathbf{h}(\mathbf{H}, \mathbf{P}, \lambda) = \mathbf{F}(\mathbf{H}, \mathbf{P}, \lambda), \tag{S26}$$

where λ is the parameter to be varied. For instance, λ could be one of the inlet pressures or inlet haematocrits. Once the solution to $\mathbf{h} = \mathbf{0}$ is known for a starting value of $\lambda = \lambda_0$, this solution is used as the starting solution for numerical continuation as λ varies.

The homotopy functions of Equations (S24-S26) are used to generate bifurcation diagrams or sets of equilibria of the network. The process of generating a bifurcation diagram or a single equilibrium is summarised by the flow chart in Figure S1. All numerical continuation is performed by the bifurcation analysis software AUTO 07p (Doedel et al, 1999).

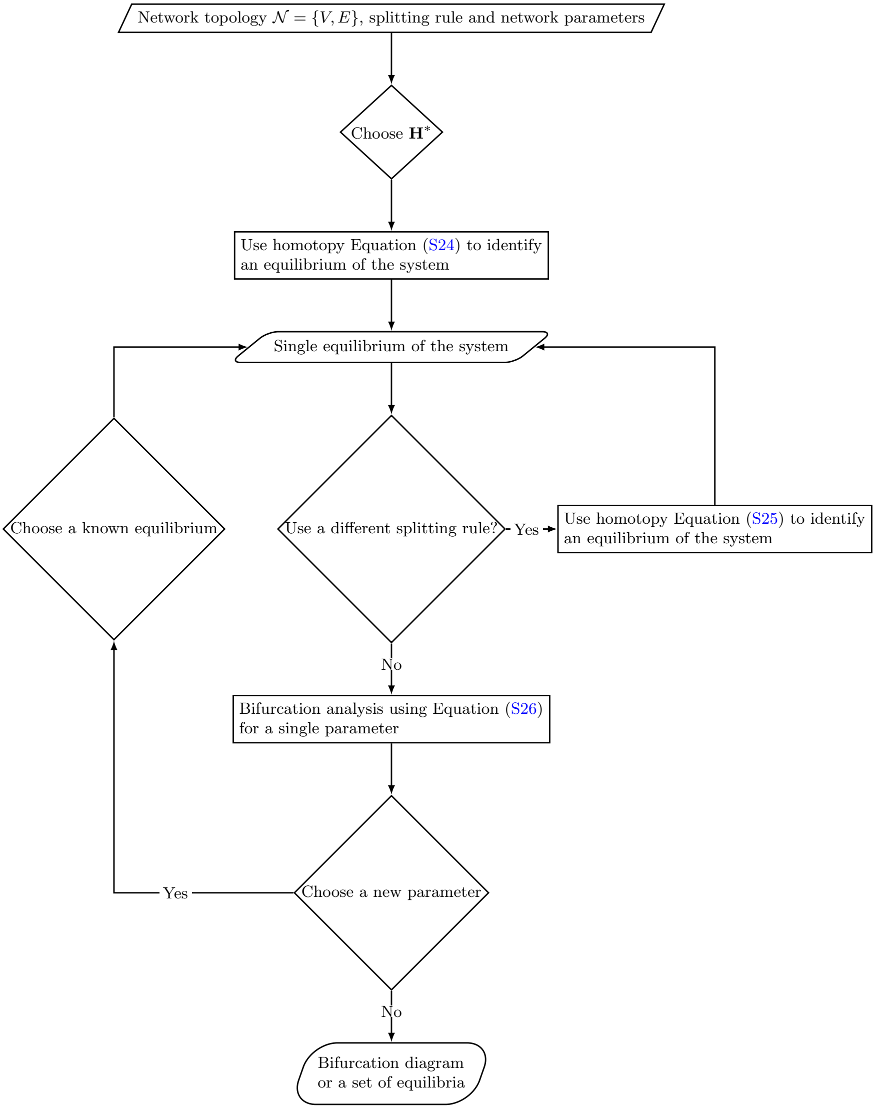

Figure S1: Flow diagram of a process to generate bifurcation diagrams for the system of network equations.

S3 Asymmetry of the haematocrit distribution in the triangle network

In this section, we investigate the contribution of plasma skimming, at bifurcation and convergence units, on the haematocrit distribution in the triangle network.

S3.1 Properties of a convergence unit

The haematocrit value in the outflowing vessel of a convergence unit in Figure 1b is lower than the haematocrit in one of the inlet vessels as a direct consequence of the network conservation laws. Rearranging the solutions to $F_{(v,w)} = 0$ for Equation (19) yields the following expression:

$$H_{(v,w)} = \frac{Q_{(u,v)}}{Q_{(v,w)}} H_{(u,v)} + \frac{Q_{(z,v)}}{Q_{(v,w)}} H_{(v,z)}, \tag{S27}$$

using equation (18), this expression simplifies to the following form:

$$\underbrace{\frac{Q_{(u,v)}}{Q_{(v,w)}}}_{=r} H_{(u,v)} + \left(1 - \frac{Q_{(u,v)}}{Q_{(v,w)}}\right) H_{(v,z)} = H_{(v,w)}, \tag{S28}$$

which simplifies to:

$$H_{(v,w)} = rH_{(u,v)} + (1 - r)H_{(v,z)}. \tag{S29}$$

As $0 \leq r \leq 1$:

$$\min(H_{(v,z)}, H_{(u,v)}) \leq H_{(v,w)} \leq \max(H_{(v,z)}, H_{(u,v)}). \tag{S30}$$

S3.2 Properties of a bifurcation unit

Flow bifurcations have similar properties to flow convergences. Consider the bifurcation unit in Figure 1a. With $F_{(v,w)}(H, P)$ defined by the left hand side of Equation (20), if $F_{(v,w)}(H, P) = 0$ then:

$$H_{(v,w)} = \psi_{(v,w)}(\underbrace{Q_{(v,w)}/Q_{(u,v)}}_{=r}, H_{(u,v)}) \underbrace{\frac{Q_{(u,v)}}{Q_{(v,w)}}}_{=1/r} H_{(u,v)}. \tag{S31}$$

This expression simplifies to give:

$$H_{(v,w)} = \frac{1}{r}\psi_{(v,w)}(r, H_{(u,v)})H_{(u,v)}, \tag{S32}$$

such that $r = Q_{(v,w)}/Q_{(u,v)}$. This equation is important because $H_{(v,w)}$ is greater than or less than $H_{(u,v)}$ depending on the properties of $\psi_{(v,w)}$ and the value of r.

Consider the general splitting rule of Equation (13) with:

$$A = -\frac{7}{D_{(u,v)}} \frac{(D_{(v,w)} - D_{(v,z)})}{(D_{(v,w)} + D_{(v,z)})}, \rho = 2.0, X_0 = 0.05.$$

In Figure S2, we sketch the ratio $\psi_{(v,w)}(r)$ for two sets of values of the diameters for the bifurcation unit in Figure 1a. In Figure S2a, $D_{(v,w)} = D_{(v,z)}$, while in Figure S2b, $D_{(v,w)} = 100\mu\text{m}, D_{(v,z)} = 5\mu\text{m}$ and $D_{(u,v)} = 10\mu\text{m}$. If $r = 0.6$ and the diameters of the daughter vessels are equal, Figure S2a indicates that $\psi_{(v,w)}(r)/r > 1$. In contrast, if $r = 0.6$ and the diameters of the vessels are the same as those used to generate Figure S2b, then $\psi_{(v,w)}(r)/r < 1$. Therefore, the haematocrit in the daughter vessels depends on the splitting rule and the network parameters. However, if $A = 0$, then $\psi_{(v,w)}(r) \geq r$ for $r \geq 0.5$ for all values of ρ and X_0. In this case, the daughter vessel with the larger flow also has a greater haematocrit value than the parent vessel.

A similar expression can be formulated for the haematocrit in the other daughter vessel, $H_{(v,z)}$. It is straightforward to show that:

$$H_{(v,z)} = \frac{(1 - \psi_{(v,w)}(r))}{1 - r} H_{(u,v)}. \tag{S33}$$

If $\psi_{(v,w)}(r) > r$, then $1 - \psi_{(v,w)}(r) < 1 - r$ and $H_{(v,z)} < H_{(u,v)}$. We conclude, without loss of generality, that:

$$H_{(v,z)} \leq H_{(u,v)} \leq H_{(v,w)}, \tag{S34}$$

for the haematocrit values of the bifurcation unit in Figure 1a.
Another important property of splitting rules is the existence of a maximum of $\psi_{(v,w)}/r$ in Figures S2c and S2d. Both figures show the plot of:

$$f_{(v,w)}(r) = \frac{\psi_{(v,w)}(r)}{r}, \tag{S35}$$

which is also the scaling factor for the daughter haematocrit in Equation (S32). There exists a value of $r = r_1 < 1$ such that $\psi_{(v,w)}(r) = r$, because $\psi_{(v,w)}(1 - X_0) = 1$, and $1 - X_0 < 1$. Equation (S35) is once differentiable on $(0, 1)$ because $\psi_{(v,w)}(r)$ and $1/r$ are both once differentiable on $(0, 1)$. Therefore, a maximum of $f_{(v,w)}(r)$ always exists in an interval between $(r_1, 1 - X_0)$. Taking the derivative of $f_{(v,w)}(r)$ with respect to r, it is straightforward to show that:

$$f'_{(v,w)}(r) = \frac{1}{r}(\psi'_{(v,w)}(r) - f_{(v,w)}(r)). \tag{S36}$$

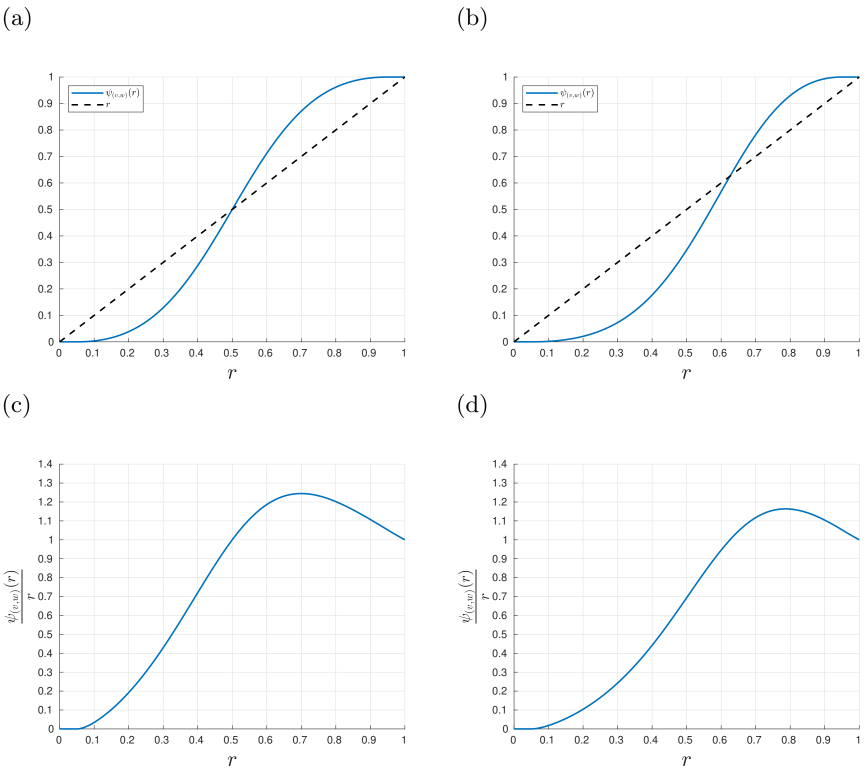

Figure S2: Comparison of $\psi_{(u,v)}$ with the two different parameter sets for the bifurcation unit in Figure 1a. (a): Plot of $\psi_{(u,v)}(r)$ for the vessel diameters $D_{(v,w)} = D_{(v,z)}$; (b): Plot of $\psi_{(u,v)}(r)$ for $D_{(v,w)} = 100\mu$m , $D_{(v,z)} = 5\mu$m and $D_{(u,v)} = 10\mu$m; (c): Plot of $\psi_{(u,v)}(r)/r$ for $D_{(v,w)} = D_{(v,z)}$; (d): Plot of $\psi_{(u,v)}(r)/r$ for $D_{(v,w)} = 100\mu$m , $D_{(v,z)} = 5\mu$m and $D_{(u,v)} = 10\mu$m.

$\psi'_{(v,w)}(r_1) > 1$, because $\psi_{(v,w)}(r)$ intersects r at $r = r_1$. Therefore:

$$f'_{(v,w)}(r_1) = \frac{1}{r_1}(\underbrace{\psi'_{(v,w)}(r_1)}_{>1} - \underbrace{f_{(v,w)}(r_1)}_{=1}) > 0. \tag{S37}$$

If we take the first derivative of Equation (13) with respect to r, we obtain the following function for the derivative:

$$\psi'_{(v,w)}(r) = \begin{cases} 0 & \text{if } r < X_0 \\ 0 & \text{if } r > 1 - X_0 \\ \frac{(1-2X_0)e^A\rho((r-X_0)(1-r-X_0))^{\rho-1}}{(e^A(r-X_0)^\rho+(1-r-X_0)^\rho)^2} & \text{if } X_0 \leq r \leq 1 - X_0 \end{cases} \tag{S38}$$

Since $\psi'_{(v,w)}(1-X_0)=0$ we have that:

$$f'_{(v,w)}(1-X_0)=\frac{1}{1-X_0}(\underbrace{\psi'_{(v,w)}(1-X_0)}_{=0}-f_{(v,w)}(1-X_0))<0. \tag{S39}$$

Since $f'_{(v,w)}(r_1)>0$ and $f'_{(v,w)}(1-X_0)<0$, at least one maximum must exist in the interval $(r_1,1-X_0)$. Furthermore, $f_{(v,w)}(r)$ eventually decreases as r approaches 1. For $r_2\in[1-X_0,1]$, due to the fact that the derivative of $\psi'_{(v,w)}(r)$ is equal to 0 for all values of r_2, we have that:

$$f'_{(v,w)}(r_2)=\frac{1}{r_1}(\underbrace{\psi'_{(v,w)}(r_2)}_{=0}-\underbrace{f_{(v,w)}(r_2)}_{>0})<0. \tag{S40}$$

Counterintuitively, this means that as the flow ratio, r, approaches 1, the haematocrit vessel (v,w) must decrease.

S3.3 Asymmetric haematocrit distribution of the triangle network

Figure S3 shows the plots of $H_{(4,6)}$ and $H_{(5,6)}$ in the triangle network as length ratio β is varied, for the same network parameters explored in Section 4.1. Note that:

$$H_{(4,6)}>\overline{H}_{(1,4)}=\overline{H}_{(2,5)}=0.45>H_{(5,6)}, \tag{S41}$$

for the $(+)$ equilibria, and the opposite is true for the $(-)$ equilibria. This inequality is a consequence of the plasma skimming properties of blood discussed in Sections S3.1 and S3.2, and the presence of the redundant vessel. The triangle network must contain one flow bifurcation, at either nodes 4 or 5. In the case of the $(+)$ equilibria, this flow bifurcation is at node 4. Therefore, as $Q_{(4,6)}>Q_{\langle 4,5\rangle}$, $H_{(4,6)}>0.45>H_{\langle 4,5\rangle}$, and $H_{(5,6)}<\max(\{H_{\langle 4,5\rangle},\overline{H}_{\langle 2,5\rangle}\})=0.45$. Therefore, the triangle network has an asymmetric haematocrit distribution for the $(-)$ and $(+)$ equilibria, even when the network is symmetric ($\beta=1$).

Although we do not include plots of the haematocrits in the fixed vessels of the extended-triangle network, we can infer a similar asymmetry of the haematocrit distribution. Asymmetry of the haematocrit distribution would also emerge as a result of non-zero flow in present in the extended-triangle network redundant vessels, and the plasma skimming properties.

S4 Effect of different splitting rules

So far our results in Section 4 have been generated using the Pries 1990 splitting rule first described by Pries et al (1990). This splitting rule is defined by Equation (13), and the coefficients in Equations (14-16). Splitting rules like

(a) (b)

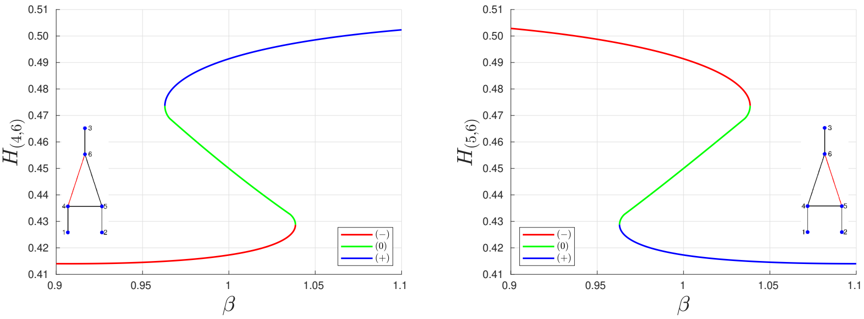

Figure S3: Series of bifurcation diagrams showing how the haematocrit in the fixed vessels of the triangle network vary as β varies. (a) haematocrit $H_{(4,6)}$. (b) haematocrit $H_{(5,6)}$.

those defined by Pries and Secomb (2005) and Klitzman and Johnson (1982) share the same form as the Pries 1990 rule but use different A, ρ and X_0 coefficients. We have demonstrated that the triangle and extended-triangle network admit multiple equilibria when the flow direction in one of the redundant vessels changes direction as one or more parameters are varied. Although these splitting rules share the same basic structure, this does not rule out the possibility that multiple equilibria are an artefact of the Pries 1990 splitting rule instead of arising from the geometric properties of the network. Therefore, in this section, we will generate bifurcation diagrams for the extended-triangle network for the splitting rule coefficients in Table S1.

Splitting rule	ρ	A	X_0
Pries 1990	$1 + 6.98\frac{1-H_{(u,v)}}{D_{(u,v)}}$	$-\frac{6.96}{D_{(u,v)}} \log\left(\frac{D_{(v,w)}}{D_{(v,z)}}\right)$	$\frac{0.4}{D_{(u,v)}}$
Pries 2005	$1 + 6.98\frac{1-H_{(u,v)}}{D_{(u,v)}}$	$-13.29\left[\frac{D^2_{(v,w)}-D^2_{(v,z)}}{D^2_{(v,w)}+D^2_{(v,z)}}\right]\frac{1-H_{(u,v)}}{D_{(u,v)}}$	$0.964\frac{1-H_{(u,v)}}{D_{(u,v)}}$
Simple I, II & III	$1.1, 1.4, 2$	$-\frac{7}{D_{(u,v)}}\frac{(D_{(v,w)}-D_{(v,z)})}{(D_{(v,w)}+D_{(v,z)})}$	0.05

Table S1: Summary of the different functional forms that are used in Equation (13) to generate the different haematocrit splitting rules. The indices of the vessels correspond to the bifurcation unit in Figure 1a and the functions of ρ, A and X_0 determine the ratio of RBCs $Q_{(v,w)}H_{(v,w)}/Q_{(u,v)}H_{(u,v)}$.

In this section, we consider five different splitting rules, two from the literature, and three that have been newly created. The existing splitting rules are the

Pries 1990 (Pries et al, 1990) and Pries 2005 (Pries and Secomb, 2005) rules; the other splitting rules are detailed in Table S1. The following parameter values are used when simulating flow in the extended-triangle network:

$$\beta = 1.1, \alpha = 0.5, D = 10, \overline{H}_{(1,7)} = \overline{H}_{(2,8)} = 0.45,$$

where α and β are defined by equations (22-24). We vary $\overline{P}_1$ and set $\overline{P}_2 = 1 - \overline{P}_1$ in this section.

Figure S4 shows the bifurcation diagrams for the flow and haematocrit variables of the extended-triangle network for the two Pries rules and the Simple I splitting rule, as $\overline{P}_1$ increases from 0.45 to 0.55. Figures S4c and S4d indicate that differences between the equilibria are most pronounced for the variables of the vessels furthest away from the inlets. Regardless of the difference in the values of $H_{\langle 4,5 \rangle}$ and $Q_{\langle 4,5 \rangle}$, the difference between equilibria for the three splitting rules is small.

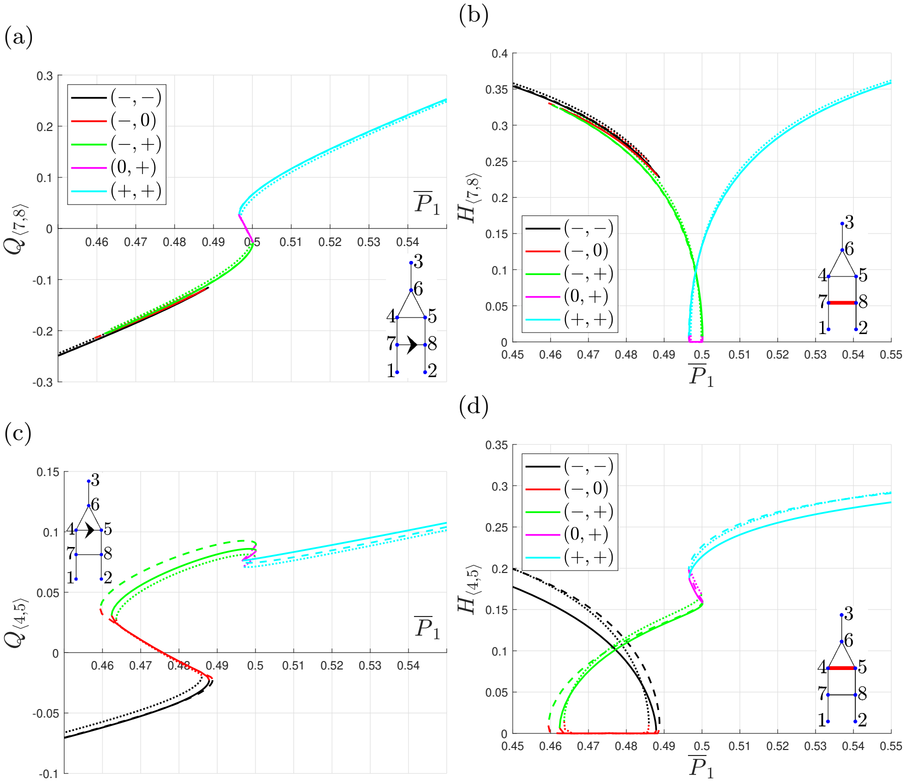

Figure S4: Series of bifurcation diagrams showing how the flow and haematocrit in the redundant vessels in the extended-triangle network change as the inlet pressure $\overline{P}_1$ varies for the Pries 1990, Pries 2005 and Simple II splitting rules. Key: each equilibrium is labelled $-, 0$ or $+$ depending on whether the flow in the redundant vessels is negative, intermediate, or positive. The arrows on the network diagrams indicate the vessels for the corresponding flow plot, and defines which flow direction is considered positive. The equilibria are represented by the different line styles: dotted line for the Pries 1990 rule, dashed line for the Pries 2005 rule, and solid line for the Simple II rule.

The bifurcation diagrams for the extended-triangle network are similar regardless of the choice of the splitting rule. The network admits a unique solution branch between $\overline{P}_1 = 0.45$ and $\overline{P}_1 \approx 0.46$ with negative flow in both redundant vessels. At $\overline{P}_1 \approx 0.46$, two more solution branches emerge from a fold bifurcation, one with positive flow in vessel $\langle 4, 5 \rangle$, and one with intermediate flow. At $\overline{P}_1 \approx 0.49$, the solution branches with intermediate and negative flow in vessel $\langle 4, 5 \rangle$ collide at another fold bifurcation. Another two solution branches emerge from a fold bifurcation at $\overline{P}_1 \approx 0.495$ with positive and intermediate flow in vessel $\langle 7, 8 \rangle$. The solution branches with negative and intermediate flow also collide at a fold bifurcation at $\overline{P}_1 \approx 0.5$, and the solution branch with positive flow in both redundant vessels exists for all

higher values of $\overline{P}_1$.

As with the bifurcation diagrams in Figure 3, the intervals of multiple equilibria exist in between two fold bifurcations, in which the direction of the flow in a redundant vessels changes direction. This first interval is approximately $0.45 \lesssim \overline{P}_1 \lesssim 0.49$ and the second interval is approximately $0.495 \lesssim \overline{P}_1 \lesssim 0.5$. The difference in sizes of the intervals for the three splitting rules are not significant. The first interval can be more clearly identified in Figures S4c and S4d, and the second interval can be more clearly identified in Figures S4a and S4b. Applying the definitions of the types of flow to both intervals, the first interval is characterised by different flow in vessel $\langle 4, 5 \rangle$, and the second by the flow in vessel $\langle 7, 8 \rangle$. Therefore, regardless of the differences between the solution branches, multiple equilibria still emerge as the flow in a redundant vessel changes direction in between two fold bifurcations.

Figure S5 shows a similar comparison to that of Figure S4. As with Figure S4, the extend-triangle network admits two intervals of multiple equilibria in Figure S5. Both of these intervals are created by the same common double fold bifurcation structure. However, the difference in size for the intervals is much larger for Figure S5. With the intervals being the smallest for the Simple I splitting rule and largest for the Simple III splitting rule. In the case of the Simple III splitting rule, the two intervals overlap slightly, creating a small interval in which five equilibria exist.

Apart from differences in the size of the intervals of multiple equilibria, all equilibria in Figures S4 and S5 have the same qualitative properties as the equilibria in Section 4: all intervals of multiple equilibria are created by the changing flow direction in a redundant vessel, and all equilibria have unique flow configurations. Therefore, we conclude that the link between the equilibria of a network and the flow in the redundant vessels is a property of the two networks regardless of the choice of splitting rule.

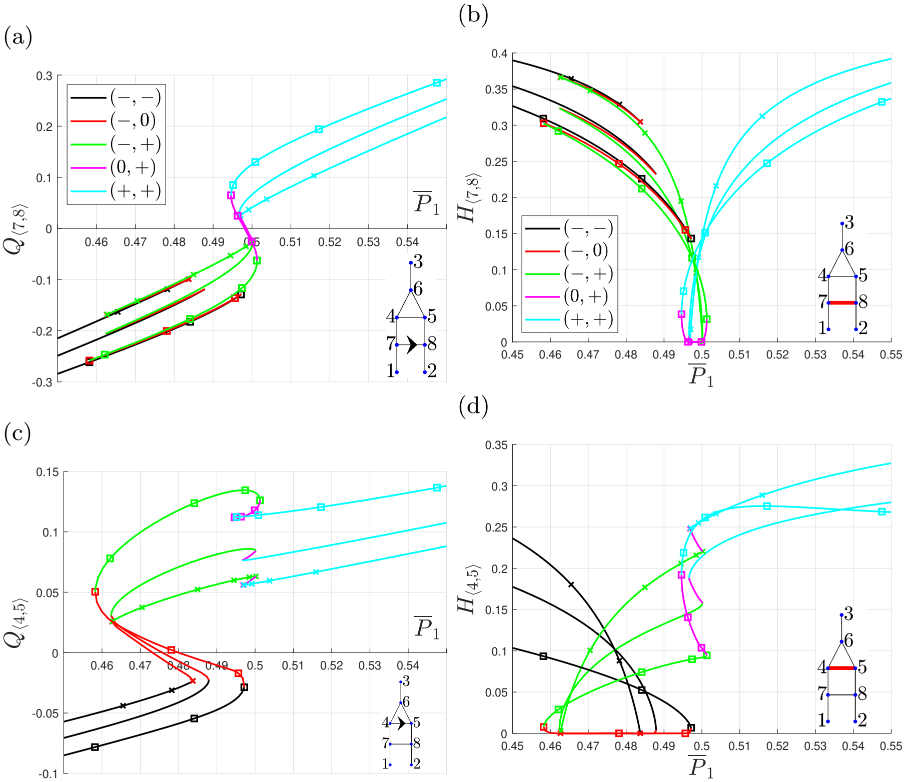

Figure S5: Series of bifurcation diagrams showing how the flow and haematocrit in the redundant vessels in the extended-triangle network change as the inlet pressure $\overline{P}_1$ varies for the three simple splitting rules. Key: each equilibrium is labelled $-, 0$ or $+$ depending on whether the flow in the redundant vessels is negative, intermediate, or positive. The arrows on the network diagrams indicate the vessels for the corresponding flow plot, and defines which flow direction is considered positive. The equilibria are represented by the different line styles: solid line with x markers for the Simple I rule, solid line for the Simple II rule, and solid line with squares for the Simple III rule.

References

Allgower EL, Georg K (2003) Introduction to numerical continuation methods, SIAM, chap 2, pp 1–2. https://doi.org/10.1137/1.9780898719154

Doedel E, Champneys A, Fairgrieve T, et al (1999) Auto 97: Continuation and bifurcation software for ordinary differential equations (with homcont)

Klitzman B, Johnson PC (1982) Capillary network geometry and red cell distribution in hamster cremaster muscle. American Journal of Physiology-Heart and Circulatory Physiology 242(2):H211–H219. https://doi.org/10.

1152/ajpheart.1982.242.2.H211

Kuznetsov YA, Kuznetsov IA, Kuznetsov Y (1998) Elements of applied bifurcation theory, vol 112. Springer, https://doi.org/10.1007/978-1-4757-3978-7

Pries AR, Secomb TW (2005) Microvascular blood viscosity in vivo and the endothelial surface layer. American Journal of Physiology-Heart and Circulatory Physiology 289(6):H2657–H2664. https://doi.org/10.1152/ajpheart.00297.2005

Pries AR, Secomb TW, Gaehtgens P, et al (1990) Blood flow in microvascular networks: Experiments and simulation. Circulation Research 67(4):826–834. https://doi.org/10.1161/01.RES.67.4.826
